# Transcriptomic and Functional Analyses of Phenotypic Plasticity in a Higher Termite, *Macrotermes barneyi* Light

**DOI:** 10.3389/fgene.2019.00964

**Published:** 2019-10-04

**Authors:** Pengdong Sun, Ganghua Li, Jianbo Jian, Long Liu, Junhui Chen, Shuxin Yu, Huan Xu, Chaoliang Lei, Xuguo Zhou, Qiuying Huang

**Affiliations:** ^1^Hubei Insect Resources Utilization and Sustainable Pest Management Key Laboratory, College of Plant Science and Technology, Huazhong Agricultural University, Wuhan, China; ^2^Marine Biology Institute, Shantou University, Shantou, China; ^3^School of Bioscience and Bioengineering, Guangdong Provincial Key Laboratory of Fermentation and Enzyme Engineering, South China University of Technology, Guangzhou, China; ^4^Department of Entomology, University of Kentucky, Lexington, KY, United States

**Keywords:** higher termites, transcriptomic analyses, gene expression, eclosion, mobility

## Abstract

Eusocial termites have a complex caste system, which leads to the division of labor. Previous studies offered some insight into the caste differentiation in lower termites; however, few studies were focusing on the molecular mechanisms of higher termites with sophisticated societies. Comparative transcriptomic analyses of five immature castes of a higher termite, *Macrotermes barneyi* Light, suggest that phenotypic plasticity is modulated by an array of transcriptional changes, including differentially expressed genes (e.g., caste-biased genes *Vtg* and *TnC*), co-expression networks (e.g., genes associated with nymph reproduction), and alternative splicing (e.g., events related to muscle development in presoldiers). Transcriptional (RT-PCR and RT-qPCR) and functional (*in vivo* RNAi) validation studies reveal multiple molecular mechanisms contributing to the phenotypic plasticity in eusocial termites. Molecular mechanisms governing the phenotypic plasticity in *M. barneyi* could be a rule rather than an exception in the evolution of sociality.

## Introduction

Phenotypic plasticity is the ability of individuals to modify their physiology, morphology and/or behavior to adapt different biotic or abiotic environments and plays a crucial role in evolution and speciation (Nijhout, 1999). A primary example of phenotypic plasticity is present in the caste differentiation and division of labor in social insects, from simple societies (i.e., subsocial) to the most complex societies (i.e., eusocial). With an identical genome, the colony members of social insects show caste-specific morphologies, behavior, and social organization, which contribute to the division of labor (Duarte et al., 2011).

Termites are diploid hemimetabolous social insects whose colonies usually contain workers, soldiers, and reproductive castes (Hughes et al., 2008). The specific phenotypes can be derived from differential gene expression and regulation (Kapheim et al., 2015; Masuoka et al., 2018). Although genes and networks involved in the caste differentiation and division of labor have been a research focus for the past decade (Poulsen et al., 2014; Scharf, 2015; Harrison et al., 2018), the molecular signatures underlying phenotypic plasticity in the higher termites are mostly lacking.

**Figure 1:**
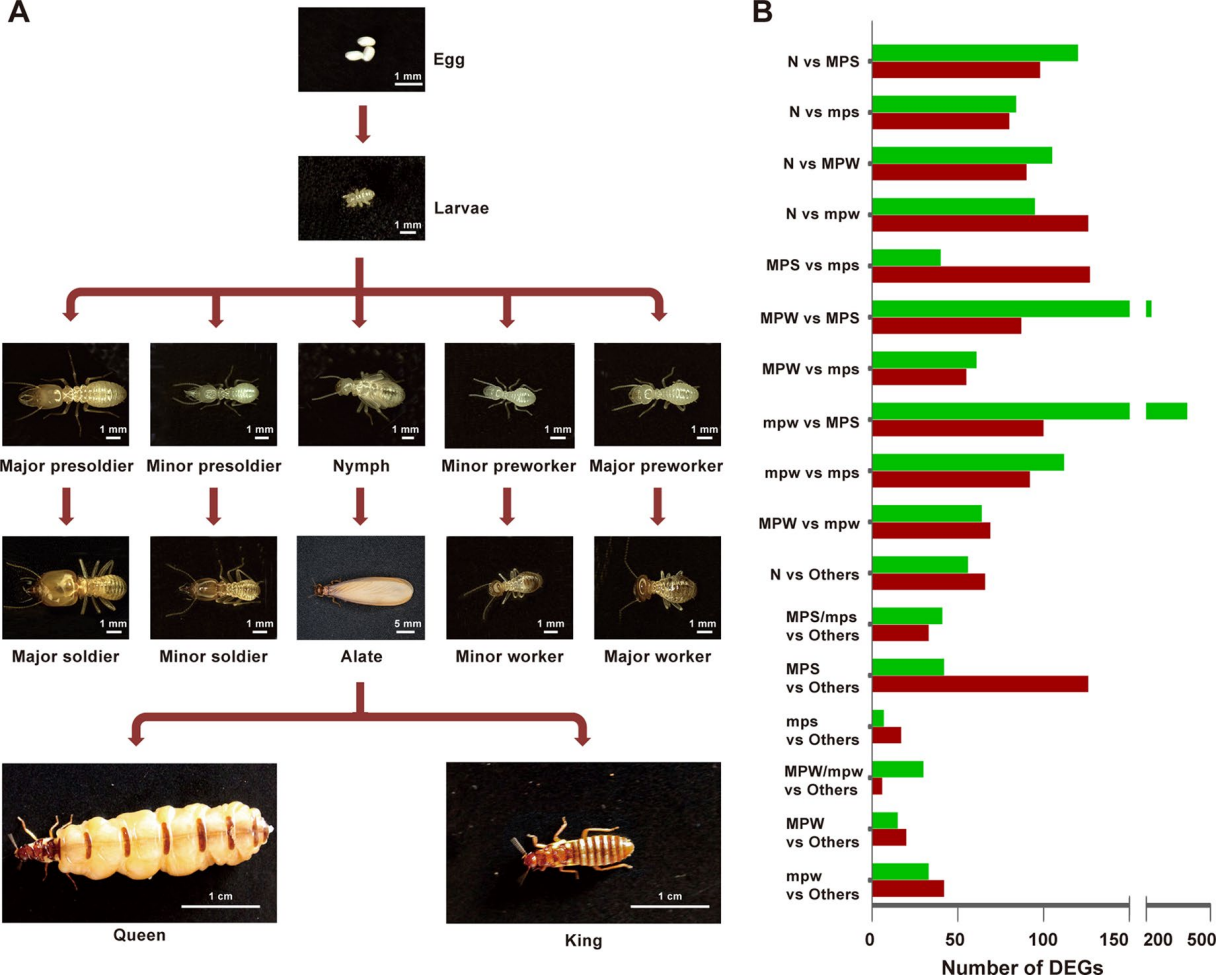
The developmental pathway and distribution of DEGs in the higher termite *M. barneyi*. **(A)** The developmental pathway of *M. barneyi*. **(B)** Distribution of DEGs from the 17 comparative groups among the five immature castes. N, nymphs; MPS, major presoldiers; mps, minor presoldiers; MPW, major preworkers; mpw, minor preworkers.

Compared to lower termite species, one character of the caste system in the higher termite *Macrotermes* species is that the caste differentiation may be irreversible (Andersson, 1984; Szathmáry and Maynard Smith, 1995), and caste differentiation pathway in *Macrotermes* species is often sex-specific, for example, the major workers are males, while the minor workers are females (Noirot, 1990). *Macrotermes barneyi*, a fungus-growing subterranean termite, contains seven mature castes, including queens, kings, alates, major and minor soldiers, and major and minor workers (**Figure 1A**). Alates, queens and kings are reproductive castes and develop from nymphs (Eggleton, 2011), while the remaining mature castes are derived from the other immature castes, including major and minor presoldiers, and major and minor preworkers (**Figure 1A**, Noirot, 1990). Major soldiers provide defense near or within the colony using their strong mandibles, and minor soldiers provide protection for the foraging workers (Thorne and Haverty, 1991; Roux and Korb, 2004; Wang et al., 2009; Li et al., 2015b). Major workers are primarily responsible for the nest construction and foraging, while minor workers carry out in-nest duties, such as feeding, nursing, and grooming (Wang et al., 2009).

To fill the current knowledge gap, we included all five immature castes, nymphs, major and minor presoldiers, major and minor preworkers, to investigate how the phenotypic plasticity generated in the immature castes of *M. barneyi*. To reach our goal, we carried out the following objectives: 1) searched for molecular signatures, including differentially expressed genes (DEGs), co-expression networks, and alternative splicing (AS), associated with caste-specific phenotypes using comparative RNA-Seq analyses; and 2) validated the RNA-Seq results at the transcriptional (RT-PCR and RT-qPCR) and functional (*in vivo* RNAi) levels.

## Materials and Methods

### Sample Collection

*M. barneyi* is a common termite species in China and collection permit is not required. *M. barneyi* field colonies were collected from Shuangfeng county, Hunan province, China (Colonies 1–5), Wuhan county, Hubei province, China (Colony 6), Changsha county, Hunan province, China (Colonies 7–9), Jian county, Jiangxi province, China (Colonies 10 and 11). GPS coordinate of each colony was listed at **Table S1**. Immature castes, including nymphs, major and minor presoldiers, and major and minor preworkers, were identified and collected based on their morphological traits (**Figure S1**; body lengths, W = 220.838, *P* < 0.001; head width, F = 1624.882, df = 4, *P* < 0.001). Caste samples were stored at -80 °C for the subsequent RNA-Seq, RT-qPCR, and AS event analyses. Due to the difficulty of collecting all five immature castes within the same colony in the field, we combined castes from different colonies for the RNA-Seq analysis. Specifically, replication 1 contained all five immature castes from Colony 1. Replication 2 included major and minor presoldiers and major and minor preworkers from Colony 2, and nymph from Colony 4. Similarly, replication 3 combined major and minor presoldiers and major and minor preworkers from Colony 3 with nymph from Colony 5 (**Table S1**). For the functional analysis of *Vtg* and *TnC*, the targeted immature castes (nymphs, minor presoldiers, and major preworkers) were collected from colonies 6–11. More details can be found in **Table S1** and **Text S1**.

### Illumina Sequencing, Read Mapping, and DEG Analysis

Whole-body samples derived from the five immature castes were used to obtain sufficient amounts of RNA for the Illumina sequencing. Three biological replications were carried out for each immature caste, and a total of 15 cDNA libraries were constructed and sequenced using Illumina HiSeq^™^ 2000. Based on the gene expression levels within the 15 cDNA libraries, we calculated the Pearson correlations and excluded colony effect. Clean reads were filtered from the raw reads after sequencing. HISAT (version: v0.1.6-beta) (Kim et al., 2015) was used to perform genome mapping with the reference genome of *M. natalensis* (Poulsen et al., 2014) as the default option. We used StringTie (Pertea et al., 2015) to reconstruct transcripts and used cuffcompare to compare reconstructed transcripts to reference annotation. CPC (CPC, http://cpc.cbi.pku.edu.cn/) were used to predict the coding potential of novel transcripts. Novel coding transcripts were merged with the reference transcripts to get a complete reference for downstream analyses. We aligned clean reads to the gene model and completed reference using BWA-MEM (Li, 2013) and Bowtie2 (Langmead and Salzberg, 2012), respectively. Based on the gene alignment, the random distribution of reads was assessed. Gene expression was calculated using RSEM (version: v1.2.12) (Li and Dewey, 2011).

DEG analyses were performed with 17 comparative groups, including 10 groups of pairwise comparisons among immature castes, and five groups of each immature caste compared with the other four immature castes, and two groups of major and minor presoldiers/preworkers compared with the other three immature castes. DEGs of 17 comparative groups were identified using Noiseq (Tarazona et al., 2015) software with parameters set at fold-change ≥ 2.0 and probability ≥ 0.8, and another software DESeq (Storey and Tibshirani, 2003) with parameters set at fold-change ≥ 2.0 and adjusted *P*-value ≤ 0.001. We then compared the DEGs identified by the software. Considering the specific caste system of *M. barneyi*, we chose nine comparative groups as the targeted groups for gene co-expression network analysis and detection of AS events. More details can be found in **Text S1**.

### Gene Co-Expression Network Analysis

Gene co-expression network analysis was performed using WGCNA (Langfelder and Horvath, 2008), and eigengenes (the average signed normalized gene expression values) were clustered and finally merged into 17 modules. To identify the modules associated with caste-specific phenotypes, we screened DEGs of the targeted groups from the genes clustered in the 17 modules. Given that only nymphs can develop into reproductive caste in higher termites, we assumed nymph “0” and other immature castes “1” to obtain a “0–1” matrix of reproductive trait for all immature castes. We correlated reproductive traits with modules, and calculated the correlation coefficient between genes and reproductive traits, and modules, respectively. If genes showed strong relationship with reproductive traits and were key genes in modules associated with reproduction, then we considered them as candidate genes for reproduction. The relationship between modules and reproductive trait was displayed through the moduleTraitCor and labelled with the Heatmap package of WGCNA (Zhang and Horvath, 2005). More details can be found in **Text S1**.

### Detection and Validation of AS Events

We carried out three replicates of RNA-Seq analysis for each of the five immature castes. rMATS (version: v3.0.9) was used to identify differential AS events between two samples (Shen et al., 2014). Binary Alignment/Map (BAM) files were outputted from the process of mapping the clean reads to the reference genome by HISAT, and were employed for the identification of AS events using rMATS. Then we ran rMATS for the nine groups of pairwise comparison (FDR ≤ 0.05). RT-PCR experiments were performed to validate the AS events detected using rMATS. Four differential AS events of four genes were selected for the validation study based on their potential relevance to the phenotypic plasticity using three biological replications. The PCR products were subjected to electrophoresis on a 2% agarose gel. Densities of AT bands were estimated by Quantity one (version 4.6.2). More details can be found in **Text S1**.

### Validation of 16 DEGs by RT-qPCR

The 16 DEGs involved in relevant biological functions were selected to validate their expression among the five immature castes by RT-qPCR. Total RNA of whole bodies from individuals of the five immature castes (colonies 1–5) were extracted by using TRIzol reagent (Ambion) according to the manufacturer’s protocol and then were treated with DNase I to remove the genomic DNA. RNA quality was calculated and checked using a Bioanalyzer 2100 algorithm (Agilent Technologies). Approximately 1 μg of RNA was converted to cDNA using the PrimeScript™ RT Reagent Kit with gDNA Eraser (Perfect Real Time) (TakaRa, Dalian, China). Based on the FPKM values, we selected a group of housekeeping genes, including *heat-shock 70 kDa protein (HSP70)*, *Glutathione-S-transferase (GST), elongation factor 1-alpha (EF1-α), glyceraldehyde-3-phosphate dehydrogenase (GAPDH)*, and *Actin*, as candidates to normalize the gene expression in RT-qPCR analysis. geNorm (Vandesompele et al., 2002) was used to evaluate the stability and the optimal number of reference genes needed for the gene expression analysis among the five immature castes (**Figure S2** and **Table S2**). The RT-qPCR analysis was performed using the My IQ^™^ Color Real-time PCR Detection System (Bio-Rad, USA) with cDNA as the template. Relative expression levels of targeted genes were calculated using the 2^-ΔΔCt^ method (Van Hiel et al., 2009) with *HSP70* and *GAPDH* as internal references. Three biological replications were set for RT-qPCR. The primers used for RT-qPCR are listed in **Table S3**.

### Preparation and Microinjection of ds*Vtg* and ds*TnC*

The dsRNAs of *Vtg* (ds*Vtg*) and *TnC* (ds*TnC*) were prepared with T7 RNA Polymerase (Thermo, MA, USA) according to the manufacturer’s instructions. Individuals of the targeted immature castes (nymphs, minor presoldiers and major preworkers) were collected from colonies 6–11 according to **Table S1**. After microinjection of ds*Vtg* and ds*TnC*, individuals of the corresponding immature castes were reared in a petri dish with a moist paper disk under laboratory conditions at a temperature of 25 ± 1 °C, 80% relative humidity and 24-h darkness. Certain numbers of mature castes were reared together with the microinjected individuals according to the similar proportion of each caste in a natural colony. More details can be found in **Text S1**.

### Quantification of RNAi Efficiency

One nymph, three minor presoldiers, and eight major preworkers were separately collected and killed in liquid nitrogen 72 h after injection of ds*Vtg* and ds*TnC*. Total RNA from the whole bodies of these individuals was extracted and treated as mentioned in the section of “Validation of 16 DEGs by RT-qPCR.” Specific primers for *Vtg* and *TnC* were designed based on their sequences in the transcriptome data (**Table S3**). Using the CFX96 Touch^™^ Real-Time PCR Detection System (Bio-Rad, USA), the expression of *Vtg* in nymphs was evaluated, and the expression of *TnC* was determined in minor presoldiers and major preworkers. Relative expression levels of *Vtg* and *TnC* were calculated using the 2^-ΔΔCt^ method (Van Hiel et al., 2009) with *HSP70* and *GAPDH* as internal references. Three biological replications were set for the RT-qPCR assay of *Vtg* and *TnC*.

### Functional Analyses of *Vtg* and *TnC* by RNAi Technology

Nymphs with similar development states were collected approximately 15 days before their eclosion. After injection of ds*Vtg*, the eclosion rate of nymphs in one petri dish was recorded every 24 h for 4 days. Four days after the ds*Vtg* injection, the body weight of nymphs and the wing size of eclosed nymphs were measured, and the morphology of ovary of eclosed nymphs were observed. A total of seven biological replications (with four nymphs in one petri dish consider a biological replication) were used in this test. The same number of nymphs treated with ds*GFP* were synchronously tested as a control.

The minor presoldiers and major preworkers were selected for the mobility test 72 h after injection of ds*TnC*. Briefly, a 6-well plate (3.5 cm in diameter) placed in a dish with moist paper was used as an arena for this test. A single minor presoldier or major preworker was moved from the treatment petri dish into the arena for 5 min of adaption before the test. Then, the 6-well plate was placed directly under a digital video camera for live video recording. Each recording lasted 5 min. The video recordings were converted to mpeg format for the mobility analysis using Noldus EthoVision-XT system (Noldus Information Technology, The Netherlands). The average velocity and the total moving distance of each minor presoldier or major preworker during the 5-min observation period were analyzed as indicators of the athletic ability. A total of 22 minor presoldiers (five individuals from colony 7 and 17 individuals from colony 8) as 22 replications and 24 major preworkers (11 individuals from colony 7 and 13 individuals from colony 8) as 24 replications were tested in this mobility assay. After the mobility assay, the individual minor presoldiers or major preworkers were killed with liquid nitrogen, and each of the two individuals were pooled together for the determination of ATP levels using a luciferase-based ATP assay kit (Beyotime, Haimen, China). There were 11 and 8 biological replications for ATP determination in minor presoldiers and major preworkers, respectively. The same number of minor presoldiers and major preworkers treated with ds*GFP* were synchronously tested as a control.

### Statistical Analysis

Statistical analysis of differential gene expression was carried out using Noiseq and DESeq, respectively. Alternative splicing events were identified using rMATS. GO and pathway enrichment was analyzed by phyper (package of R). Gene co-expression network analysis was performed using WGCNA. Other statistical analyses were conducted using IBM SPSS Statistical 18.0 software (SPSS Inc., Chicago, IL, USA). Data distribution of morphological parameters and functional analyses of *Vtg* and *TnC* were evaluated by Shapiro–Wilk test (**Table S4**), and the equality of variances were evaluated by Leven’s test. To assessing the significant differences among more than two data groups, one-way ANOVA and Tukey’s HSD were used to analyze the normally distributed data with equal variances, while Welch’s ANOVA and Games–Howell test were used to evaluate the normally distributed data with unequal variances (**Table S5**). For significant difference analyses between two data groups, a paired Student’s t-test was used to assess the normally distributed data with equal variances, while the Mann–Whitney U-test was used to assess the abnormally distributed data (**Table S5**).

### Availability of Supporting Data and Materials

The datasets supporting the results of this article are included in the article and its supplementary materials. The raw sequence data for the 15 transcriptomes of *M. barneyi* from Illumina sequencing are available in the NCBI Short Read Archive (accession number: SRP056611).

## Results and Discussion

The five immature castes (nymphs, major and minor presoldiers, major and minor preworkers) can be distinguished by their specific morphologies or distinctly different sizes (**Figure S1**). Whole-body samples derived from the five immature castes of *M. barneyi* were deeply sequenced to generate more than 70 Gb of high-quality clean reads (Q20 > 96.19%, GC contents ranged from 42.88% to 45.85%, **Table S6**). We then aligned clean reads to *M. natalensis* genome (Poulsen et al., 2014); ∼60% and ∼43% of the mapped rate was aligned with the reference genome and genes, respectively, which were balanced among the 15 transcriptomes (**Table S7**). An average of 46,969 transcripts was identified for each immature caste, which could be aligned to an average of 30,027 genes (**Table S7**). The read distributions on genes were uniformly (**Figure S3**). Pearson correlations for all of the 15 libraries of five immature castes indicated the reliability of our experimental results (**Figure S4**).

DEGs identified by Noiseq were almost all included in DEGs identified by DESeq (**Data S1**). To reduce the false positives, we selected Noiseq for the subsequent DEG analyses. Among the 17 comparative groups of the five immature castes, the comparison groups of minor preworkers (mpw) vs major presoldiers (MPS) and major preworkers (MPW) vs MPS showed the most-abundant DEGs (492 and 312, respectively, **Figure 1B**). DEGs in these two groups could be mainly clustered to GO terms associated with cuticle structure, antioxidant and peroxidase activity (**Table S8**). Followed by these two groups, the comparison groups of nymphs (N) vs mpw and N vs MPS also showed particularly abundant DEGs (221 and 218, respectively, **Figure 1B**), which were mainly clustered in cuticle structure, muscle development and energy metabolism (**Table S8**).

### Gene Co-Expression Modules Associated With Caste-Specific Phenotypes

**Figure 2:**
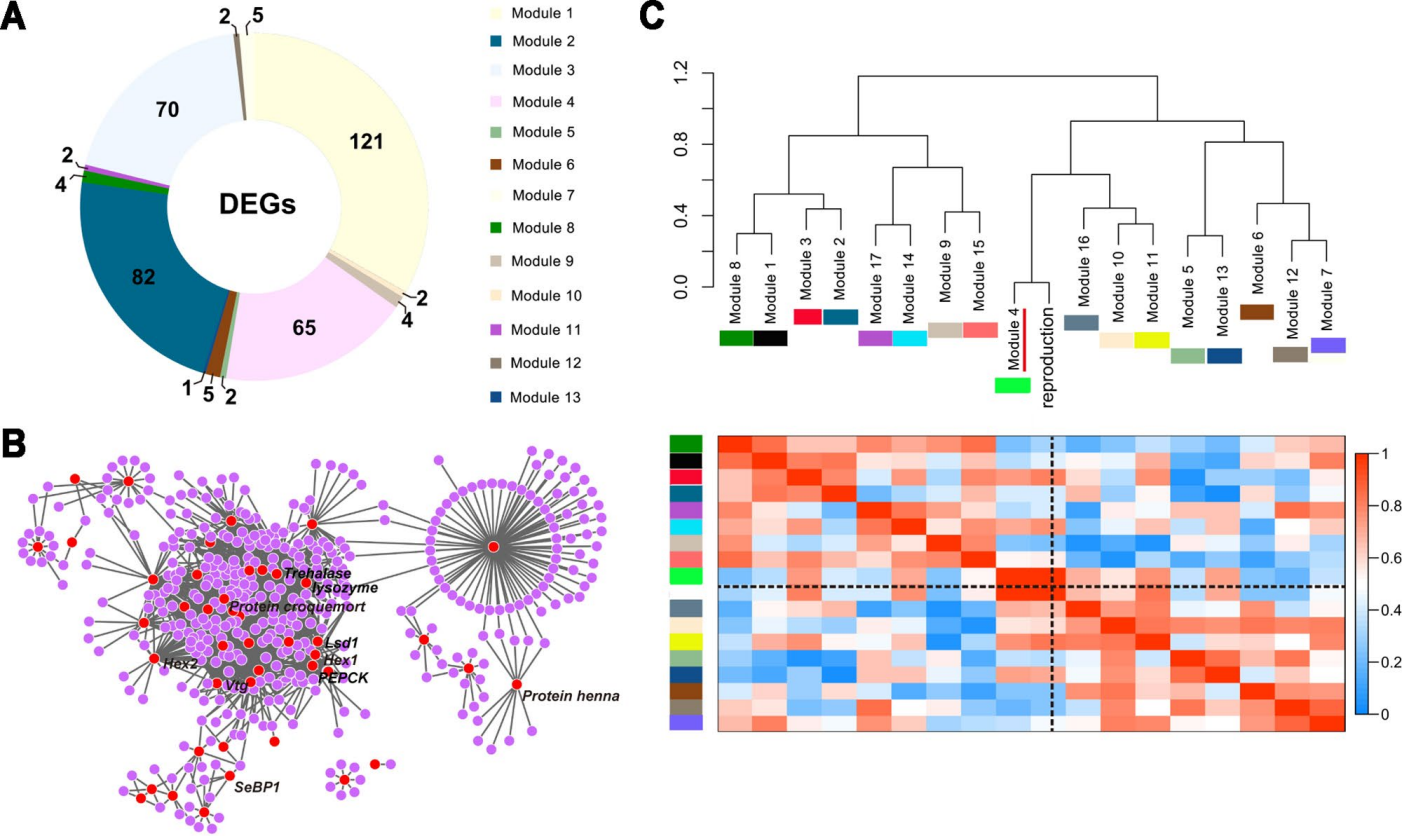
Gene co-expression modules among the five immature castes. **(A)** DEGs among the five immature castes are non-randomly distributed across 13 of 17 modules. The different colors of the regions correspond to the different modules. **(B)** Graphical representation of the co-expression network of module 4. Each dot indicates a gene, and each edge indicates a potential interaction between the two genes. DEGs are showed as red dots, and the names of genes involved in nymph-specific phenotypes are labelled. **(C)** Correlation of expression patterns in the 17 identified modules with the reproduction. Colors in the heatmap are scaled with the value of correlation coefficients indicated by the scale bar on the right side. A dendogram showing the relationship between the modules and reproduction is at the top of the heatmap, and each module is indicated by the colored square under the module name and y-axis. The black dotted lines indicate the region of reproduction in the heatmap. Module 4 which was tightly associated with the reproduction-associated phenotypes of nymphs is indicated with the red line.

We identified 17 modules by weighted gene co-expression network analysis (WGCNA) (Zhang and Horvath, 2005; Langfelder and Horvath, 2008), and each module represented a set of co-expressed or interacting genes (**Figure S5**). The DEGs of the nine targeted comparative groups were organized among 13 modules, and the top four containing the most DEGs were modules 1, 2, 3, and 4 (**Figure 2A**). Among these four modules, module 4 was clustered by DEGs mainly from the comparison of nymph vs other immature castes (**Table S9**). Furthermore, the DEGs in module 4, especially genes associated with the specific phenotypes of nymphs [such as *Hexamerin1* (*Hex1*), *Hexamerin2* (*Hex2*), *vitellogenin* (*Vtg*), *Lipid storage droplets surface-binding protein 1* (*Lsd1*), etc.], were well-connected in the co-expression network (**Figure 2B**). Considering the potency of nymphs to develop into reproductive castes, we further took reproduction as the external information to correlate with the 17 modules. Module 4 was identified to be the module most closely associated with reproduction (**Figure 2C**).

### AS Events Involved in Caste-Specific Phenotypes

To explore the relationship of AS events and caste-specific phenotypes in *M. barneyi*, we identified more than 30,000 AS events basing on the nine targeted comparative groups (**Table S10** and **Text S1**). The most-abundant differentially spliced genes (78) with 122 AS events were identified in presoldiers (FDR ≤ 0.05, **Table S11**). Notably, muscle development genes (22) with 63 AS events comprised a larger proportion of differentially spliced genes in presoldiers (**Table S12**). Many AS events (106) were also identified in preworkers (FDR ≤ 0.05, **Table S11**), and approximately a half of AS events (48) were associated with muscle development (**Table S12**). However, nymphs exhibited particularly few differentially regulated AS events compared to those of the other immature castes (FDR ≤ 0.05, **Tables S10** and **S11**).

**Figure 3:**
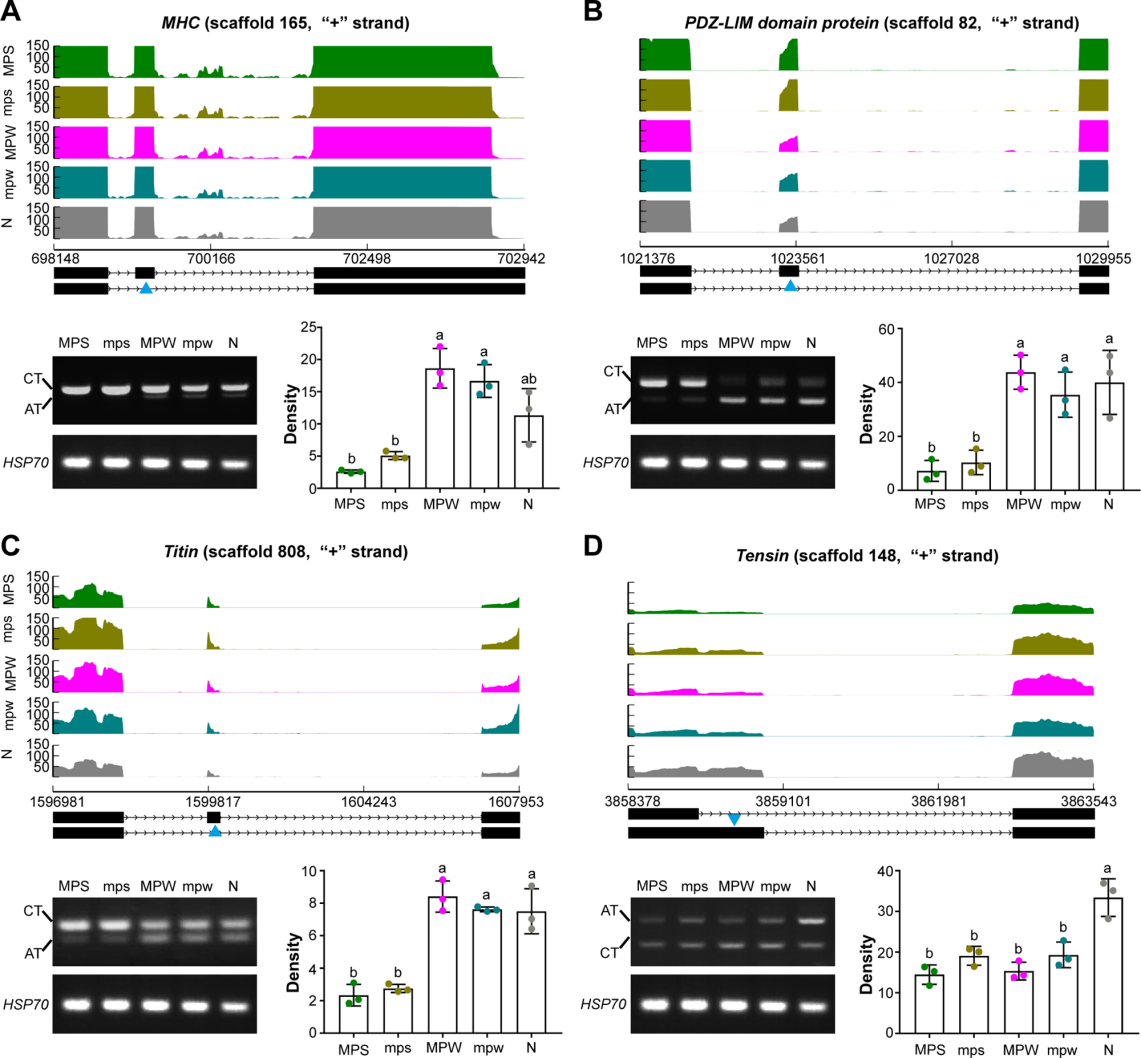
The four differentially spliced muscle genes among the five immature castes. SE events of **(A)** *MHC*, **(B)** *PDZ-LIM domain protein* and **(C)** *Titin*, and **(D)** A5SS event of *Tensin* were identified by RNA-Seq analysis and validated by RT-PCR. In the graphic representation of Sashimi plots, the y-axis refers to per-base expression, and the x-axis refers to genomic coordinates. The quantified mRNA isoforms are shown on the bottom (exons in black, introns as lines with arrow-heads), and blue triangles indicate the regions undergoing AS events. Changes in CTs and ATs among the five immature castes were analyzed by RT-PCR (left bottom). Densities of ATs (right bottom) were calculated from bands in the electrophoretogram at the left bottom, and the different lowercase letters over the bars denote significant differences in density of AT bands among the five immature castes (*P* < 0.05). AT, alternative transcript; CT, constitutive transcript, N, nymphs; MPS, major presoldiers; mps, minor presoldiers; MPW, major preworkers; mpw, minor preworkers.

Considering the potential significance of AS events to the muscle development in presoldiers and preworkers, we selected four genes to assess the relative abundance of alternative transcript (AT) and constitutive transcript (CT) among the five immature castes (**Table S13**). Three skipped exon (SE) events of three muscle genes, *myosin heavy-chain* (*MHC*, George et al., 1989; Hastings and Emerson, 1991), *PDZ–LIM domain protein* (Vallenius et al., 2004), and *Titin* (Lange et al., 2005), were differentially spliced between presoldiers and the other three immature castes. One alternative 5’ splicing site (A5SS) event of a muscle gene *Tensin* (Ishii and Lo, 2001) was differentially spliced between the four immature castes (presoldiers and preworkers) and nymph. RT-PCR results showed that the alternative transcripts of three SE events in *MHC* (**Figure 3A**; F = 21.950, df = 4, *P* < 0.001), *PDZ–LIM domain protein* (**Figure 3B**; F = 15.599, df = 4, *P* < 0.001) and *Titin* (**Figure 3C**; F = 38.150, <br/>df = 4, *P* < 0.001) were significantly downregulated in presoldiers in comparison to the other three immature castes. Meanwhile, alternative transcript of the A5SS event in *Tensin* was significantly downregulated in presoldiers and preworkers (**Figure 3D**; F = 18.532, df = 4, *P* < 0.001). The combined results suggested that the muscle development in presoldiers and preworkers might be regulated by the AS events.

### Molecular Signatures of Nymph-Specific Phenotypes

**Figure 4:**
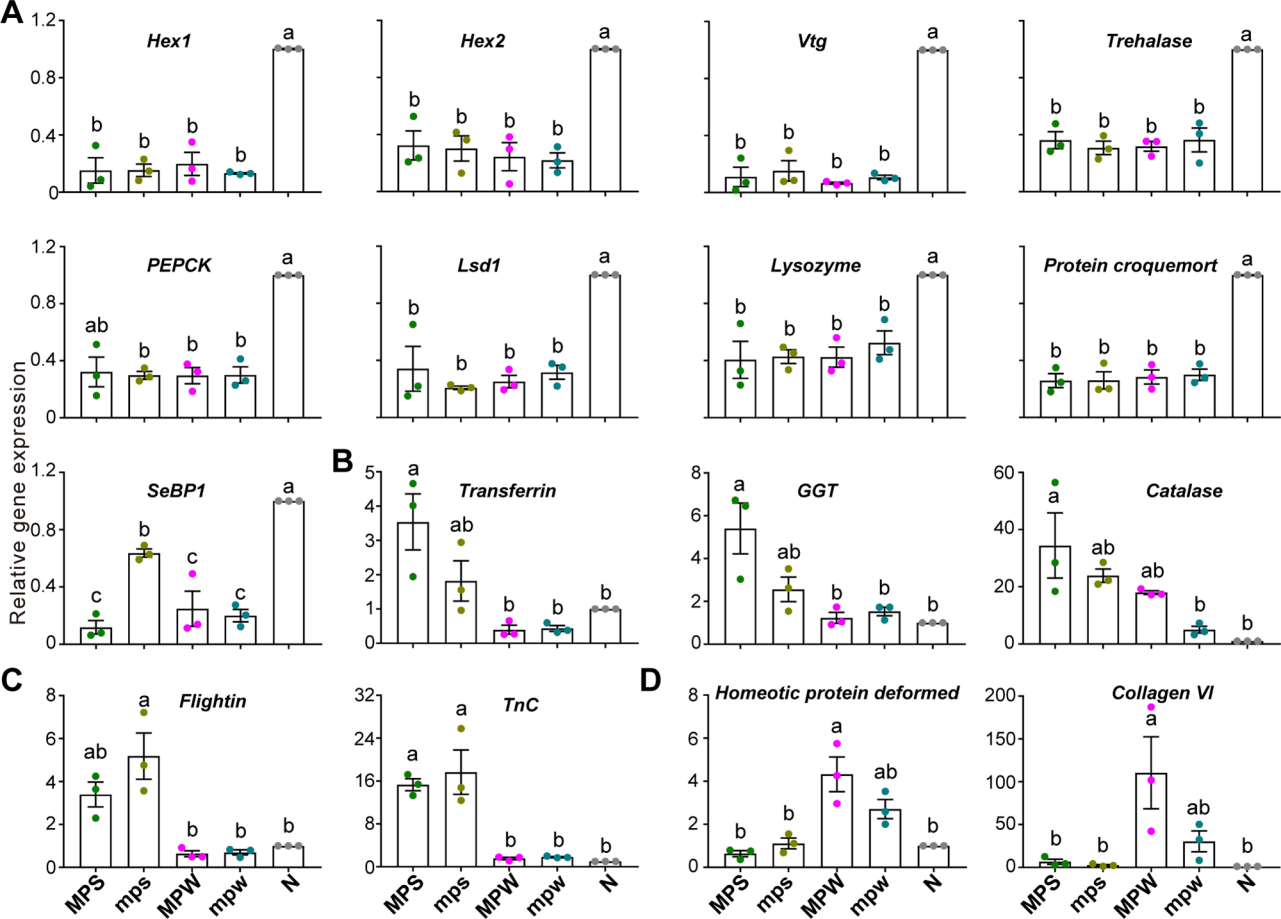
Validation of the 16 differentially expressed genes. RT-qPCR validation of the gene expression of **(A)** nine nymph-biased genes associated with caste differentiation, reproduction, energy metabolism, immune and detoxification, **(B)** three major presoldier-biased genes associated with immunity and antioxidation, **(C)** two presoldier-biased genes associated with muscle development, and **(D)** two preworker-biased genes associated with morphological development and muscle development. Error bars represent the mean value ± S.E.M. Different lowercase letters over the bars denote significant differences (*P* < 0.05). N, nymphs; MPS, major presoldiers; mps, minor presoldiers; MPW, major preworkers; mpw, minor preworkers.

We identified 66 upregulated genes and 56 downregulated genes in the group of nymph vs the other immature castes (**Figure S6A**). Similar to *Zootermopsis nevadensis* (Terrapon et al., 2014), the considerably elevated expression of immune- and detoxification-associated genes, *Protein croquemort*, *Selenium-binding protein 1* (*SeBP1*), *Protein henna*, *Lysozyme*, *epoxide hydrolase 4* (*EH4*), in nymphs (**Figures S6D** and **S6E**; *Protein croquemort*, F = 21.027, df = 4, *P* < 0.001; *SeBP1*, W = 6.742, *P* = 0.044; *Protein henna*, W = 15.793, *P* = 0.006; *Lysozyme*, F = 12.062, df = 4, <br/>*P* = 0.001; *EH4*, W = 78.634, *P* < 0.001) suggested a higher level of stress resistance and a greater investment in the protection of reproductive castes in *M. barneyi*. Three energy-related genes, *Trehalase*, *Phosphoenolpyruvate carboxykinase* (*PEPCK*) and *Lsd1*, were also highly expressed in nymphs (**Figure S6F**; *Trehalase*, W = 24.990, *P* = 0.003; *PEPCK*, F = 90.934, df = 4, *P* < 0.001; *Lsd1*, W = 6.614, *P* = 0.035). Alates demand substantial amount of energy for flights and other tasks after dispersal (Li et al., 2015a), and overexpression of energy-associated genes might contribute to the energy reservation in nymph. Six of these genes were validated by RT-qPCR to be significantly upregulated in nymphs (**Figure 4A**; *Trehalase*, F = 31.477, df = 4, *P* < 0.001; *PEPCK*, F = 26.939, df = 4, *P* < 0.001; *Lsd1*, W = 808.460, *P* < 0.001; *Lysozyme*, W = 44.380, *P* = 0.001; *Protein croquemort*, F = 51.998, df = 4, *P* < 0.001; *SeBP1*, W = 146.748, *P* < 0.001).

The transcriptomic and RT-qPCR results showed that the expression of *Hex1* and *Hex2* significantly increased in nymphs (**Figure 4A**: *Hex1*, W = 4014.291, *P* < 0.001; *Hex2*, F = 17.621, df = 4, *P* < 0.001; and **Figure S6B**: *Hex1*, W = 12.408, *P* = 0.011; *Hex2*, F = 16.495, df = 4, *P* < 0.001). These results supported *Hexamerins* as a negative regulator for caste differentiation in *M. barneyi*, which is consistent with the findings in the lower termites (Zhou et al., 2006a; Zhou et al., 2006b; Zhou et al., 2006c). The similarity in the expression of *Hexamerin* genes in preworkers and presoldiers in this study was different from the lower termites (Scharf et al., 2005), which might suggest a different mechanism governing phenotypic plasticity in higher termites.

Transcriptomic and RT-qPCR results showed that *Vtg* as a dominant egg yolk protein (Sappington and Raikhel, 1998) had the highest expression level in nymphs (**Figure 4A**: W = 3498.434, *P* < 0.001; and **Figure S6C**: F = 16.895, df = 4, *P* < 0.001). In a lower termite, *Reticulitermes flavipes*, *Vtg* showed elevated expression in presoldiers and therefore, was considered a legitimate juvenile hormone-binding protein to protect JH III from enzymatic degradation and facilitate the transport of hydrophobic JH III to its target tissues (Scharf et al., 2005). However, the expression of *Vtg* in both presoldiers and preworkers of *M. barneyi* was significantly lower than that in nymphs (**Figure 4A** and **Figure S6C**). These results supported the hypothesis that Vtg is repetitively co-opted across different termite species to serve diverse functions in different castes (Weil et al., 2007).

**Figure 5:**
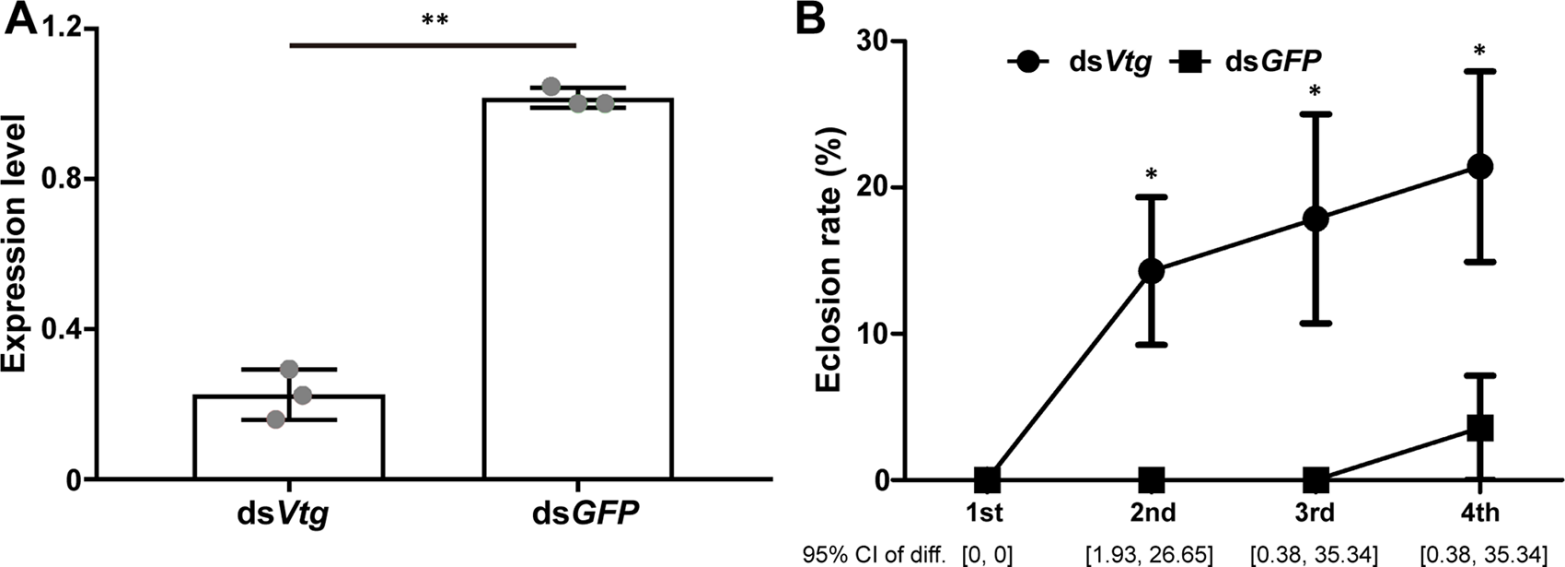
Eclosion of nymphs can be influenced by the expression of *Vtg*. **(A)** The expression level of *Vtg* after RNAi treatment. **(B)** The change in eclosion rate in nymphs during 4 days after the knockdown of *Vtg*. Data are shown as the mean ± S.E.M. *0.05 > *P* > 0.01; **0.01 > *P* > 0.001, and the 95% confidence intervals of differences of eclosion rates between ds*Vtg* and ds*GFP* treated nymph are shown in the bottom of (B).

Subsequently, we further explored the role of *Vtg* in the reproductive development of nymphs by RNAi technology. After RNAi treatment, the expression of *Vtg* was significantly downregulated in the treatment groups compared with that in the control groups (**Figure 5A**; t = -15.128, df = 2, *P* = 0.004). The eclosion of nymphs occurred earlier in the treatment groups than in the control groups, and the eclosion rates of nymphs in the treatment groups were significantly higher than in the control groups on the 2nd, 3rd, and 4th days (**Figure 5B**; 2nd day, W = 52.000, *P* = 0.025; 3rd day, W = 52.000, *P* = 0.027; 4th day, W = 51.500, *P* = 0.042). A previous study found that knockdown of *Vtg* could lead to a significantly elevated JH titer (Guidugli et al., 2005). The JH titer can be dramatically elevated just prior the eclosion (Bownes and Rembold, 1987). Thus, we suspect that a significant knockdown of *Vtg* may result in an elevated JH titer in nymphs, which signals the activation of the eclosion of nymphs in *M. barneyi*. Otherwise, there were no significant differences of the body weight of nymphs (**Figure S7A**), wing size of ecolsed nymphs (**Figure S7B**), and the ovary morphology of nymphs between the treatment groups and the control groups.

### Molecular Signatures of Sterile Caste-Specific Phenotypes

We identified 126 upregulated genes and 42 downregulated genes in the group of major presoldier vs the other immature castes (**Figure S8A**). Our results showed that the four genes associated with immunity (*Transferrin*, *gamma*-*glutamyltransferase* (*GGT*), *Lactadherin*, and *WAP four*-*disulfide core domain protein* (*WFDC*)), two genes related to antioxidation (*Catalase* and *Peroxidase*) and one gene involved in detoxification [*Flavin*-*containing monooxygenase* (*FMO*)] exhibited significantly higher expression in major presoldiers than in the other immature castes (**Figures S8B-S8D**; *Transferrin*, F = 14.718, df = 4, *P* < 0.001; *GGT*, W = 37.923, *P* = 0.001; *Lactadherin*, F = 10.618, df = 4, *P* = 0.012; *WFDC*, F = 12.015, df = 4, *P* = 0.002; *Catalase*, W = 33.484, *P* = 0.002; *Peroxidase*, F = 6.165, df = 4, *P* = 0.009; *FMO*, F = 17.416, df = 4, *P* < 0.001). Additionally, we also used RT-qPCR to further verify that the highest expression levels of the three genes occurred in major presoldiers (**Figure 4B**; *Transferrin*, W = 12.913, *P* = 0.015; *GGT*, F = 8.912, df = 4, *P* = 0.002; *Catalase*, W = 130.521, *P* < 0.001). Major soldiers are in short supply and expensive for *M. barneyi* colonies (Eggleton, 2011). The major presoldier-biased expression of genes associated with immunity, antioxidation and detoxification suggests that major presoldiers might invest more in innate immunity to the benefit of reinforcing the defensive role of major soldiers.

We identified 33 upregulated genes and 41 downregulated genes in the group of major and minor presoldiers vs the other immature castes (**Figure S9A**). The four muscle genes (*Flightin*, *Troponin C* (*TnC*), *Acylphosphatase*-*like protein*, and *SET* and *MYND domain*-*containing protein 4* (*SMYD4*)) showed significantly higher expression in presoldiers (**Figure S9C**; *Flightin*, F = 133.639, df = 4, *P* < 0.001; *TnC*, W = 76.144, *P* < 0.001; *Acylphosphatase*-*like protein*, W = 490.824, *P* < 0.001; *SMYD4*, W = 20.650, *P* = 0.003). The expression of the two genes (*Flightin* and *TnC*) was further verified by RT-qPCR (**Figure 4C**; *Flightin*, W = 7.639, *P* = 0.037; *TnC*, W = 42.770, *P* = 0.002). The higher expression levels of these muscle genes benefit the development of stronger mandibles compared to that in the other immature castes. We speculate that the defensive functions in soldiers are facilitated by the elevated expression of muscle genes at the immature stages.

A total of 6 upregulated genes and 30 downregulated genes were identified in the group of major and minor preworkers vs the other immature castes (**Figure S9B**). The four genes (*Homeotic protein deformed*, *Collagen VI*, *Pro*-*resilin* and *Cuticle protein 8*) exhibited significantly increased expression in preworkers (**Figures S9D-S9F**; *Homeotic protein deformed*, F = 42.987, df = 4, *P* < 0.001; *Collagen VI*, W = 76.767, *P* < 0.001; *Pro*-*resilin*, F = 15.376, df = 4, *P* < 0.001; *Cuticle protein 8*, W = 64.288, *P* < 0.001), and two of these genes (*Homeotic protein deformed* and *Collagen VI*) were verified by RT-qPCR (**Figure 4D**; *Homeotic protein deformed*, F = 12.844, df = 4, *P* = 0.001; *Collagen VI*, F = 10.579, df = 4, *P* = 0.013). In termites, mouth parts are usually degenerate in soldiers, while these parts are strong in workers to support their feeding behavior (Cribb et al., 2008; Toga et al., 2013). Homeotic protein deformed is crucial for the development of the maxillary and mandible (Regulski et al., 1987), and it is likely that the increased expression of this gene may contribute to the development of mouth parts in preworkers. The muscle gene, *Collagen VI*, and two cuticle genes, *Pro*-*resilin* and *Cuticle protein 8*, may be responsible for the wear resistance of leg joints or fulcral arms to facilitate workers to carry out daily tasks (Guan et al., 2006; Wang et al., 2009; Michels et al., 2016).

**Figure 6:**
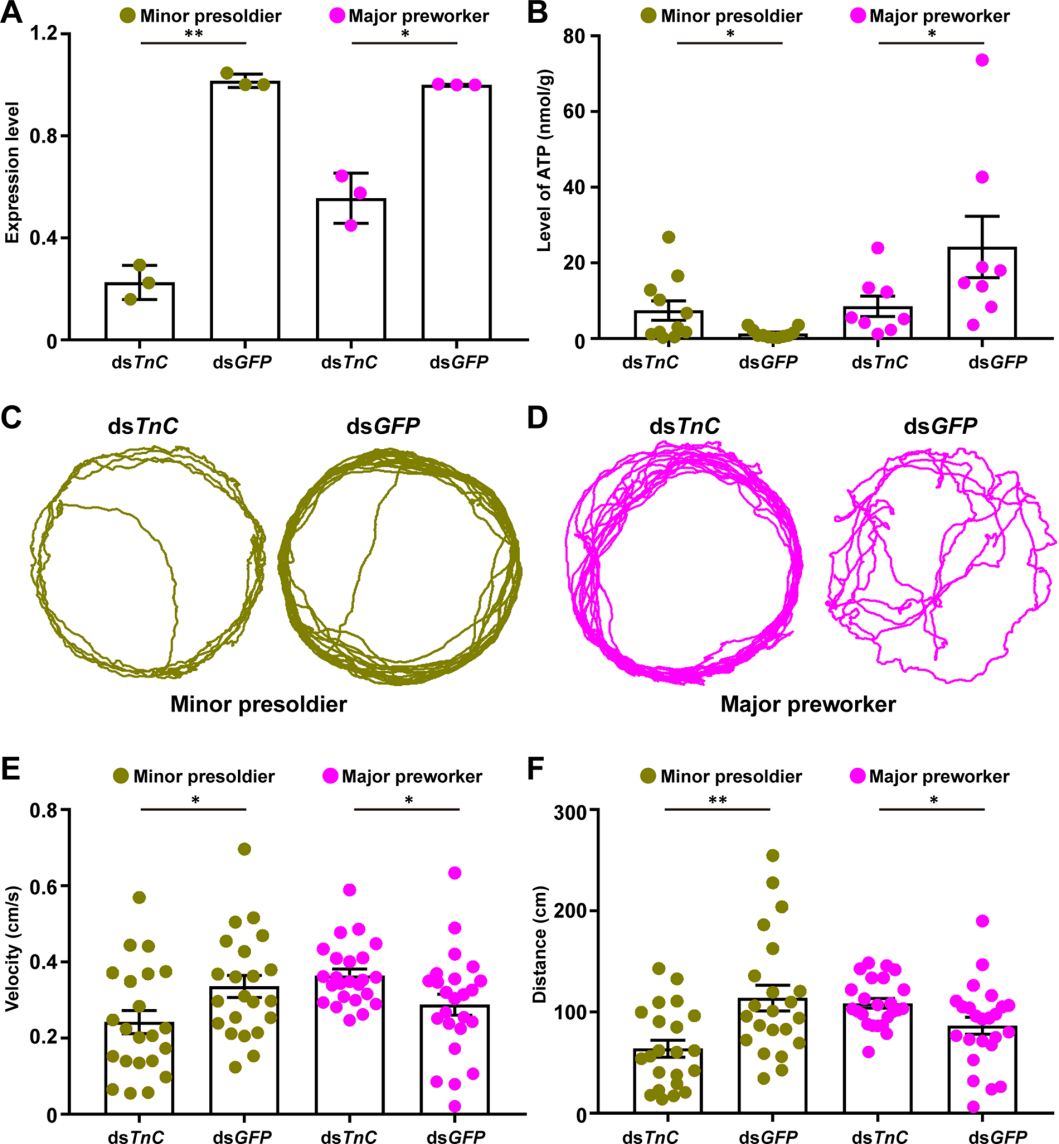
Mobility of minor presoldiers and major preworkers can be affected by the expression of *TnC*. **(A)** The expression level of *TnC* after RNAi treatment. **(B)** The ATP level after knockdown of *TnC* in minor presoldiers and major preworkers. Representative images of the motion trail after knockdown of *TnC* in **(C)** minor presoldiers and **(D)** major preworkers. **(E)** The velocities and **(F)** distances after knockdown of *TnC* in minor presoldiers and major preworkers. Data are shown as the mean ± S.E.M. *0.05 > *P* > 0.01; **0.01 > *P* > 0.001.

TnC is a signal receptor for Ca^2+^, which is released in response to electrical depolarization of muscle cells (Michels et al., 2016). The Ca^2+^-TnC complex can further bind to Troponin I (TnI) to release the activity of ATPase and regulate the skeletal muscle contraction (Parmacek and Leiden, 1991). Considering that *TnC* gene was upregulated approximately 20-fold in both major and minor presoldiers in comparison to the other three immature castes (**Figure 4C** and **Figure S9C**), RNAi was employed to study the impact of *TnC* on the mobility of minor presoldiers (representing *TnC* upregulated immature castes) and major preworkers (representing *TnC* downregulated immature castes), respectively. After RNAi treatment, the expression of *TnC* was significantly downregulated in minor presoldiers and major preworkers (**Figure 6A**; t = -12.125, df = 2, *P* = 0.007). The knockdown of *TnC* significantly increased the ATP level but decreased the velocity and distance in minor presoldiers (**Figures 6B–6C, 6E–6F**; ATP, W = 95.000, *P* = 0.039; velocity, t = -2.658, df = 21, *P* = 0.015; distance, W = 372.000, *P* = 0.004). In contrast, a statistically significant silencing of *TnC* significantly reduced the ATP level but increased the velocity and distance in major preworkers (**Figures 6B–6C, 6E–6F**; RNAi efficiency, t = -7.798, df = 2, *P* = 0.016; ATP, W = 49.000, *P* = 0.046; velocity, t = 2.362, df = 23, *P* = 0.027; distance, t = 2.450, df = 23, *P* = 0.022). In minor presoldiers, knockdown of *TnC* might lead to a reduced formation of the Ca^2+^-TnC-TnI complex and further induce an inhibition of ATP hydrolysis, which could result in ATP accumulation (Syska et al., 1976; Cachia et al., 1986). The reduction in Ca^2+^-TnC-TnI complex levels and decrease in energy supply further caused a decline in their mobility (Adelstein and Eisenberg, 1980). In major preworkers, knockdown of *TnC* might act as a signal to promote the interaction of other Ca^2+^-binding proteins, such as Calmodulin, with Ca^2+^ and TnI to generate a complex contributing to skeletal muscle contraction (Keller et al., 1982), which helps to improve the mobility of major preworkers. Additionally, extensive movements resulted in more ATP consumption in major preworkers. Collectively, *in vivo* RNAi-based functional validation study suggested the locomotion of minor presoldiers could be regulated by *TnC*, while the locomotion of minor preworkers might be regulated by the other Ca^2+^-binding proteins (e.g., Calmodulin).

## Author Contributions

PS, XZ, and QH conceived and designed the experiments. PS, GL, LL, SY, HX, and QH performed the experiments. PS, XZ, and QH analyzed the data. PS, XZ, and QH wrote the paper. All authors approved the final manuscript. All authors contributed to manuscript revision, and read and approved the submitted version.

## Funding

This research was funded by the National Natural Science Foundation of China (grant numbers: 31572322 and 31601891) and the Fundamental Research Funds for the Central Universities (grant number: 2662016PY062).

## Conflict of Interest

The authors declare that the research was conducted in the absence of any commercial or financial relationships that could be construed as a potential conflict of interest.
