# Supplementary material for: Transcriptomic and Functional Analyses of Phenotypic Plasticity in a Higher Termite, Macrotermes barneyi Light
Source: Front Genet. 2019 Oct 4;10:964. doi: 10.3389/fgene.2019.00964 (PMC6797822; doi:10.3389/fgene.2019.00964)
Supplement: Supplementary file 2 [file Table_2.docx]

**Text S1. Supplementary materials and methods**

**Sample collection**

The five colonies (Colonies 1-5) of *Macrotermes barneyi* were collected from Shuangfeng county, Hunan province, China. Five immature castes (nymphs, major and minor presoldiers, and major and minor preworkers) were identified and photographed under a stereomicroscope with a transmitted light base (Olympus SZX16). Nymphs can be identified by their distinct morphological characteristics (wing buds), and the other four immature castes can be identified by their distinct sizes with measurement by a digital imaging system (Figure S1). The five immature castes were strictly selected from the five colonies and were killed in liquid nitrogen. The whole-bodies of termite samples were stored at -80°C for the following experiments: Illumina sequencing, RT-qPCR validation of DEGs, and RT-PCR validation of alternative splicing. (see Table S1 for more details). The other six colonies (colonies 6-11) of *M. barneyi* were collected from three areas of China to carry out functional analyses of *Vtg* and *TnC* by RNAi technology (see Table S1 for more details).

**Illumina sequencing and data filtering**

Total RNA of whole bodies from individuals of the five immature castes (nymphs, major and minor presoldiers, and major and minor preworkers) were extracted using TRIzol reagent (Ambion) according to the manufacturer’s protocol and then treated with DNase I to remove genomic DNA. RNA quality was calculated and checked using the Bioanalyzer 2100 algorithm (Agilent Technologies). Total RNA samples with sufﬁcient quality (OD260/280 values between 1.8 and 2.0) were used for the subsequent analyses. Three biological replications for each immature caste were used for the Illumina sequencing. A total of 10 μg of a high-quality total RNA sample of each replication was used for the library preparation by an Illumina mRNA-Seq sample preparation kit. Briefly, mRNA was isolated and purified from total RNA using Sera-Mag Magnetic Oligo (dT) Beads, and then fragmented by divalent cations under increasing temperatures. In this study, RNAs of prokaryotic endosymbionts from *M. barneyi*, which have no poly (A) tails at the 3’end, can be filtered out from the total RNA using Sera-Mag Magnetic Oligo (dT) Beads. First-strand cDNA synthesis was reverse transcriptase with random hexamer primers using fragmented mRNA templates, and second strand cDNA was synthesized in a reaction system consisting of buffer, dNTPs, RNaseH, and DNA templates. After adenylating the 3’ ends for ligation with the Illumina adapters, the cDNA templates were puriﬁed in a gel for a size selection of approximately 250 bp and ampliﬁed via PCR to generate the ﬁnal RNA-Seq libraries. The integrity and quality of the libraries were conﬁrmed using the Agilent 2100 Bioanalyzer (Agilent Technologies). Finally, a total of 15 cDNA libraries from the five immature castes were sequenced using Illumina HiSeq^TM^ 2000.

After sequencing, the reads that satisfied any of the following conditions were removed using SOAPnuke: (i) contained adaptors, (ii) contained unknown nucleotides making up more than 5% of the sequence, or (iii) had ≥ 20% of the reads possessing a low-quality value (if the percentage of low-quality bases was over 50% in a read, the bases with sequencing quality ≤ 10 were defined as the low-quality bases). Clean reads filtered from the raw reads were used in the subsequent analyses.

**Identification and annotation of** **novel transcripts**

After mapping clean reads to the genome of *M. natalensis*, we selected the listed four class code types as novel transcripts: (i) unknown, intergenic transcript; (ii) a transfrag falling entirely within a reference intron; (iii) generic exonic overlap with a reference transcript; (iv) potentially novel isoform (fragment), with at least one splice junction shared with a reference transcript. CPC (CPC, http://cpc.cbi.pku.edu.cn/) was used to predict the coding potential of novel transcripts. Novel transcripts were used to search public databases, including the Nr, the GO database and the KEGG database.

**Annotation of DEGs**

The identified DEGs were mapped to each term of the GO database using the Blast2GO program (Conesa et al., 2005) and WEGO software (Ye et al., 2006). Next, we adopted phyper (Package of R) to perform function enrichment of DEGs. The calculated *P*-value was subjected to Bonferroni correction, and GO terms with a corrected *P*-value ≤ 0.05 were defined as significantly enriched in the DEGs. Pathway enrichment analysis was performed for DEGs compared with the whole background genome. Pathways with *Q*-values (calculated after the hypergeometric test to correct the *P*-value) ≤ 0.001 were defined as significantly enriched pathways. The striking character of the caste system of the higher termite *M. barneyi* is that soldiers are differentiated into major and minor soldiers and workers are differentiated into major and minor workers. Thus, we chose the following nine comparative groups for the next analyses of gene co-expression network and AS events: major presoldiers vs other immature castes, minor presoldiers vs other immature castes, major preworkers vs other immature castes, minor preworkers vs other immature castes, nymphs vs other immature castes, major and minor presoldiers vs other immature castes, major and minor preworkers vs other immature castes, major presoldiers vs minor presoldiers, and major preworkers vs minor preworkers.

**Gene co-expression network analysis**

Gene co-expression network analysis was performed using WGCNA (Langfelder and Horvath, 2008). WGCNA does not use DEGs to infer modules, so modules correlated with immature castes were inferred without prior knowledge about the immature caste-biased expression patterns. Genes clustered in the same module can be assumed to have some common roles in a specific pathway. In this study, only genes with expression above 0.5 FPKM across all five immature castes were selected for analysis, and the average signed normalized gene expression values were defined as eigengenes. Highly correlated eigengenes (pairwise correlation larger than 0.75) were clustered in a tree and finally merged into 17 modules. Networks were exported using the WGCNA R function exportNetworkToCytoscape, and Cytoscape was used for visualization of networks. Genes that were differentially expressed across immature castes and highly connected in module could be predicted as the drivers of that module that may putatively affect the phenotypic development. To identify modules associated with caste-specific phenotypes, we further screened DEGs clustered in the 17 modules.

**Detection and validation of AS**

Differential AS events were analyzed using rMATS (version: v3.0.9) based on the nine comparative groups. Briefly, rMATS identifies differential AS events by calculating the inclusion isoform and skipping isoform from RNA-Seq data with replications while controlling for changes in the overall gene expression levels among samples (Shen et al., 2014). Here, the process for identifying AS events can be illustrated by an example of skipped exon (SE) event. The exon inclusion level was represented by the count of uniquely mapped reads to the intron retention isoform or exon skipping isoform. The count of reads mapped to intron retention and exon skipping isoform was represented by I and S, respectively. The effective length of exon inclusion and exon skipping isoforms was denoted as lI and lS, respectively. After normalization by effective length, the exon inclusion level ψ could be calculated by ψ = (I/ll)/(I/ll +S/ls), and the proportion of reads from the exon inclusion isoform should be p = llψ/[llψ+ ls(1-ψ)]. Differential SE events were filtered out using rMATS according to the FDR ≤ 0.05 criterion.

After screening, four AS events from four genes (*MHC*, *PDZ-LIM domain protein*, *Titin* and *Tensin*) were further validated by RT-PCR using three biological replications. Total whole-body RNA from individuals of the five immature castes (colonies 1-5) was extracted and treated as mentioned above. RT-PCR experiments were performed with the cDNAs as templates. The cycling conditions were 98°C for 3 min, followed by 34 cycles of a 10-sec denaturation at 98°C, a 15-sec annealing at 54-57°C and a 15-sec extension at 72°C. The forward and reverse primers were designed based on the upstream and downstream AS regions, respectively (Table S3). The amount of the first-strand cDNA used for the RT-PCR was determined by the expression of *HSP70* and *GAPDH*. PCR products were subjected to electrophoresis on a 2% agarose gel. Densities of AT bands were estimated by Quantity One (version 4.6.2). After purification (Promega, Madison, WI, USA), PCR products were cloned into pMD 18-T Vector (TakaRa, Dalian, China), and the insert DNA was sequenced by Tsingke Biological Technology (Beijing, China).

**Preparation of ds*Vtg* and ds*TnC***

The dsRNAs of *TnC* (ds*TnC*) and *Vtg* (ds*Vtg*) were prepared with T7 RNA Polymerase (Thermo, MA, USA) according to the manufacturer’s instructions. Briefly, PCR was carried out using the composite RNA sample (derived from the five immature castes) as a template in combination with specific primers (with T7 promoter at 5` end) designed based on the sequences of target genes. The PCR products were subjected to 1% agarose gel electrophoresis, excised and purified with the Qiagen QIAquick PCR Puriﬁcation Kit (Qiagen, DER). With the purified products as templates, T7 RNA Polymerase was used in transcription reactions to generate dsRNA. The dsRNA was dissolved in DEPC-treated water and quantiﬁed by the NanoDrop 2000 Spectrophotometer (Thermo, MA, USA).

**Microinjection of ds*Vtg* and ds*TnC***

We selected nymphs as the targeted immature caste for analyzing *Vtg* function. We chose minor presoldiers and major preworkers as the two targeted immature castes for investigating *TnC* function. Individuals of the three targeted immature castes were collected from colonies 6-11 according to Table S1. Referring to the previously described method (Davis, 1987), 6 μg of ds*Vtg* was microinjected into the side of the thorax of nymph, and the different dosages of ds*TnC* were separately microinjected into the side of the thorax of minor presoldiers (3 μg) and major preworkers (1.5 μg) using a Nanoliter 2010. The corresponding immature castes injected with the same dosage of ds*GFP* were used as a control.

A certain number of mature castes were cultivated together with the microinjected immature individuals to achieve a proportion of each caste similar to that in a natural colony. For the functional analyses of *Vtg,* four treated nymphs were reared together with nine minor workers, five major workers and two minor soldiers (Video S1). For the functional analyses of *TnC,* five treated minor presoldiers were cultivated together with 10 minor workers and five major workers (Video S2), and 10 treated major preworkers were reared together with eight minor workers and two minor soldiers (Video S3).

**References**

Conesa, A., Gotz, S., Garcia-Gomez, J. M., Terol, J., Talon, M., and Robles, M. (2005). Blast2GO: a universal tool for annotation, visualization and analysis in functional genomics research. *Bioinformatics* 21, 3674-3676. doi: 10.1093/bioinformatics/bti610

Davis, N. T. (1987). Neurosecretory neurons and their projections to the serotonin neurohemal system of the cockroach *Periplaneta americana* (L.), and identification of mandibular and maxillary motor neurons associated with this system. *J. Comp. Neurol.* 259, 604-621. doi: 10.1002/cne.902590409

Langfelder, P., and Horvath, S. (2008). WGCNA: an R package for weighted correlation network analysis. *BMC Bioinformatics* 9, 559. doi: 10.1186/1471-2105-9-559

Shen, S., Park, J. W., Lu, Z. X., Lin, L., Henry, M. D., Wu, Y. N., et al. (2014). rMATS: robust and flexible detection of differential alternative splicing from replicate RNA-Seq data. *Proc. Natl. Acad. Sci. U.S.A.* 111, E5593-5601. doi: 10.1073/pnas.1419161111

Van Hiel, M. B., Van Wielendaele, P., Temmerman, L., Van Soest, S., Vuerinckx, K., Huybrechts, R., et al. (2009). Identification and validation of housekeeping genes in brains of the desert locust *Schistocerca gregaria* under different developmental conditions. *BMC Mol. Biol.* 10, 10. doi: 10.1186/1471-2199-10-56

Ye, J., Fang, L., Zheng, H. K., Zhang, Y., Chen, J., Zhang, Z. J., et al. (2006). WEGO: a web tool for plotting GO annotations. *Nucleic Acids Res.* 34, W293-W297. doi: 10.1093/nar/gkl1031
