# Supplementary material for: Transcriptomic and Functional Analyses of Phenotypic Plasticity in a Higher Termite, Macrotermes barneyi Light
Source: Front Genet. 2019 Oct 4;10:964. doi: 10.3389/fgene.2019.00964 (PMC6797822; doi:10.3389/fgene.2019.00964)
Supplement: Supplementary file 6 [file DataSheet_1.zip › Data Sheet 1/Supplementary Figures and Tables/Figrue S1.docx]

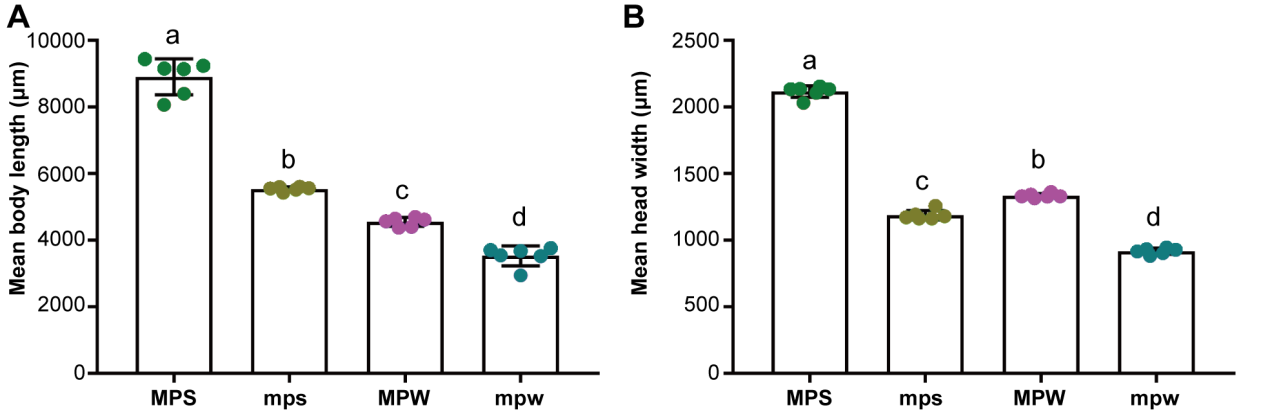


**Figure S1. Morphological parameters of four immature castes of *M. barneyi*.** **(A)** Body lengths and **(B)** head widths of four immature castes. Error bars represent the mean ± S.E.M. Different lowercase letters over the bars denote significant differences (*P* < 0.05). MPS, major presoldiers; mps, minor presoldiers; MPW, major preworkers; mpw, minor preworkers.
