# Supplementary material for: Transcriptomic and Functional Analyses of Phenotypic Plasticity in a Higher Termite, Macrotermes barneyi Light
Source: Front Genet. 2019 Oct 4;10:964. doi: 10.3389/fgene.2019.00964 (PMC6797822; doi:10.3389/fgene.2019.00964)
Supplement: Supplementary file 6 [file DataSheet_1.zip › Data Sheet 1/Supplementary Figures and Tables/Figure S2.docx]

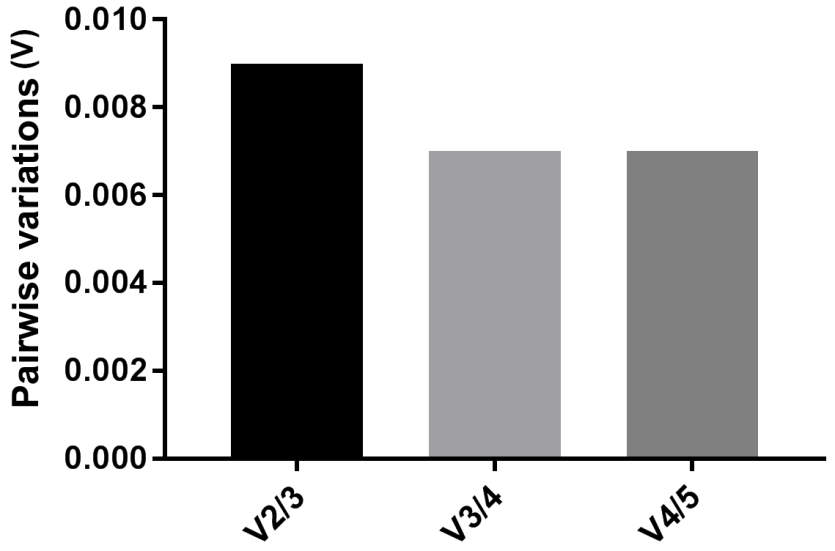


**Figure S2. GeNorm pairwise variation (V) analysis to determine the optimal number of reference genes for normalization in RT-qPCR reaction suitable for the gene expression analyses among the five immature castes of *M. barneyi***. The geNorm pairwise variation values were all below the cutoff value of 0.15.
