# Supplementary material for: Transcriptomic and Functional Analyses of Phenotypic Plasticity in a Higher Termite, Macrotermes barneyi Light
Source: Front Genet. 2019 Oct 4;10:964. doi: 10.3389/fgene.2019.00964 (PMC6797822; doi:10.3389/fgene.2019.00964)
Supplement: Supplementary file 6 [file DataSheet_1.zip › Data Sheet 1/Supplementary Figures and Tables/Figure S3.docx]

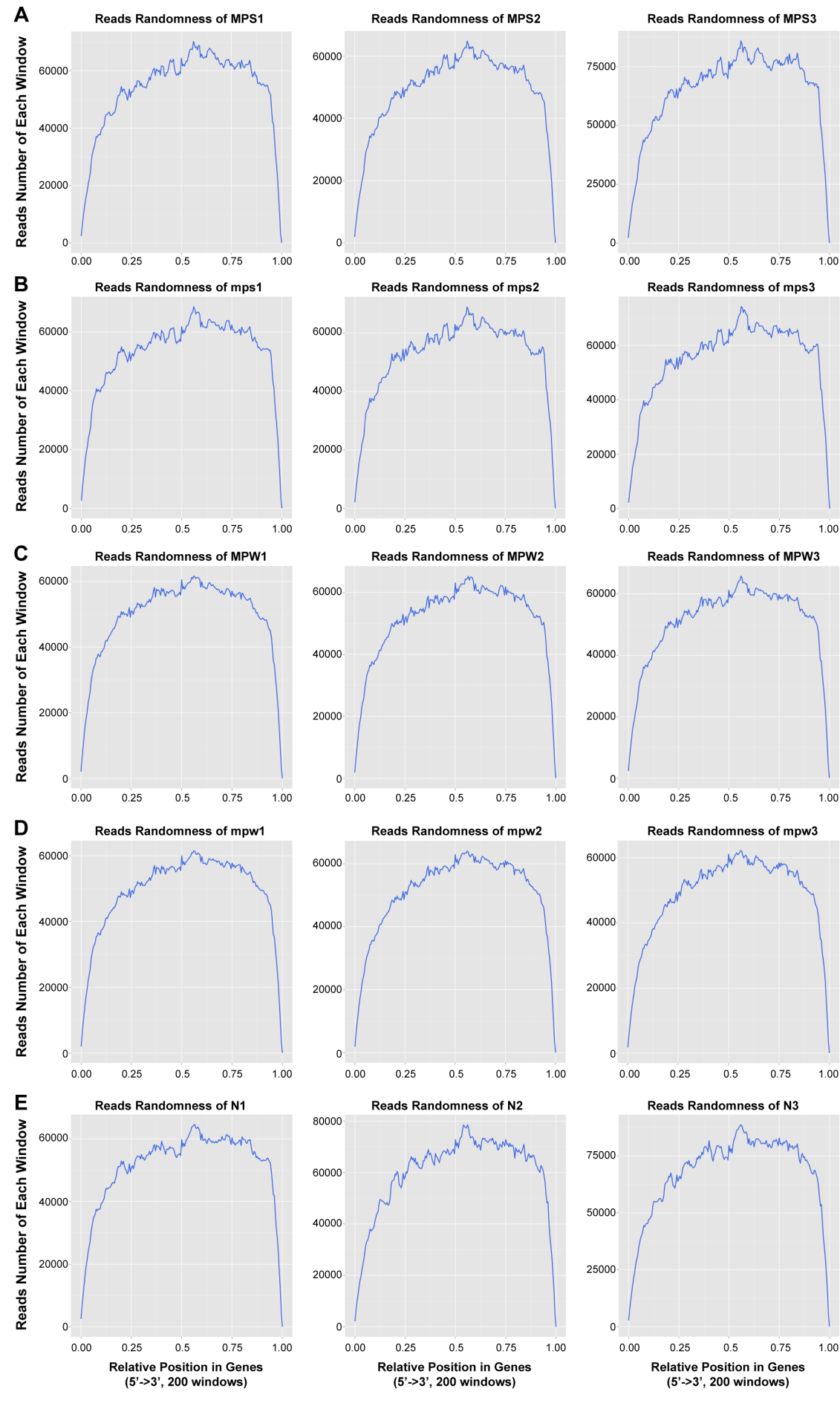


**Figure S3. Read distributions of 15 transcriptomes of *M. barneyi* based on genes of *M. natalensis*. (A)** Major presoldiers; **(B)** minor presoldiers; **(C)** major preworkers; **(D)** minor preworkers; **(E)** nymph. X-axis indicates relative position in genes, and Y-axis indicates number of reads. N, nymphs. MPS, major presoldiers; mps, minor presoldiers; MPW, major preworkers; mpw, minor preworkers.
