# Supplementary material for: Transcriptomic and Functional Analyses of Phenotypic Plasticity in a Higher Termite, Macrotermes barneyi Light
Source: Front Genet. 2019 Oct 4;10:964. doi: 10.3389/fgene.2019.00964 (PMC6797822; doi:10.3389/fgene.2019.00964)
Supplement: Supplementary file 6 [file DataSheet_1.zip › Data Sheet 1/Supplementary Figures and Tables/Figure S5.docx]

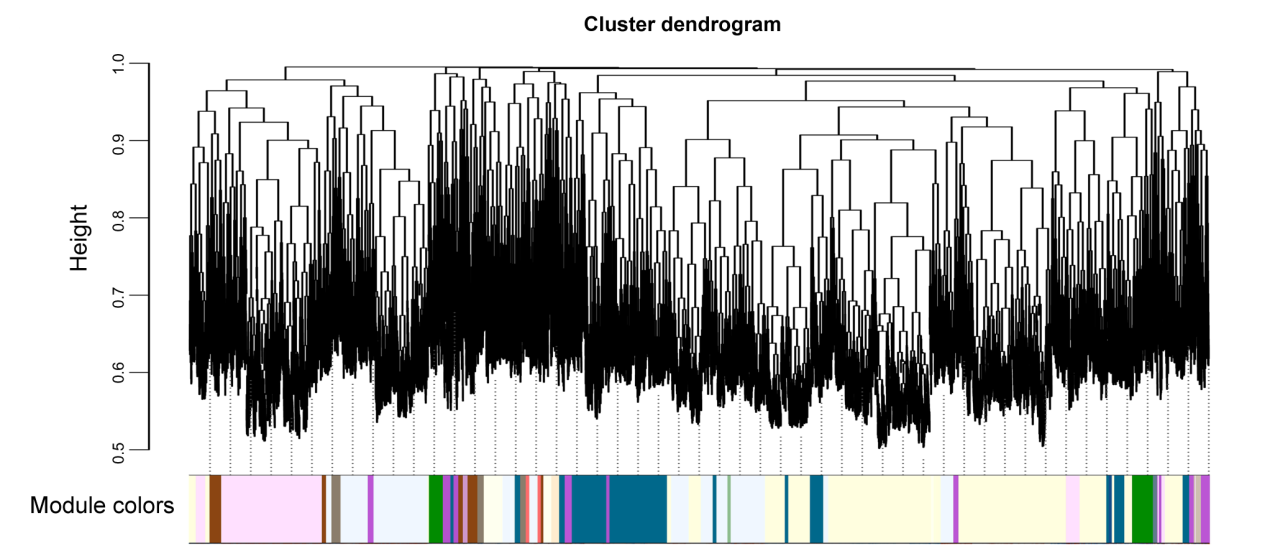


**Figure S5. Hierarchical cluster dendrogram classifies genes with FPKM > 0.5 into different co-expression modules using WGCNA.** A total of 17 major modules are indicated as colored boxes.
