# Supplementary material for: Transcriptomic and Functional Analyses of Phenotypic Plasticity in a Higher Termite, Macrotermes barneyi Light
Source: Front Genet. 2019 Oct 4;10:964. doi: 10.3389/fgene.2019.00964 (PMC6797822; doi:10.3389/fgene.2019.00964)
Supplement: Supplementary file 6 [file DataSheet_1.zip › Data Sheet 1/Supplementary Figures and Tables/Figure S6.docx]

**
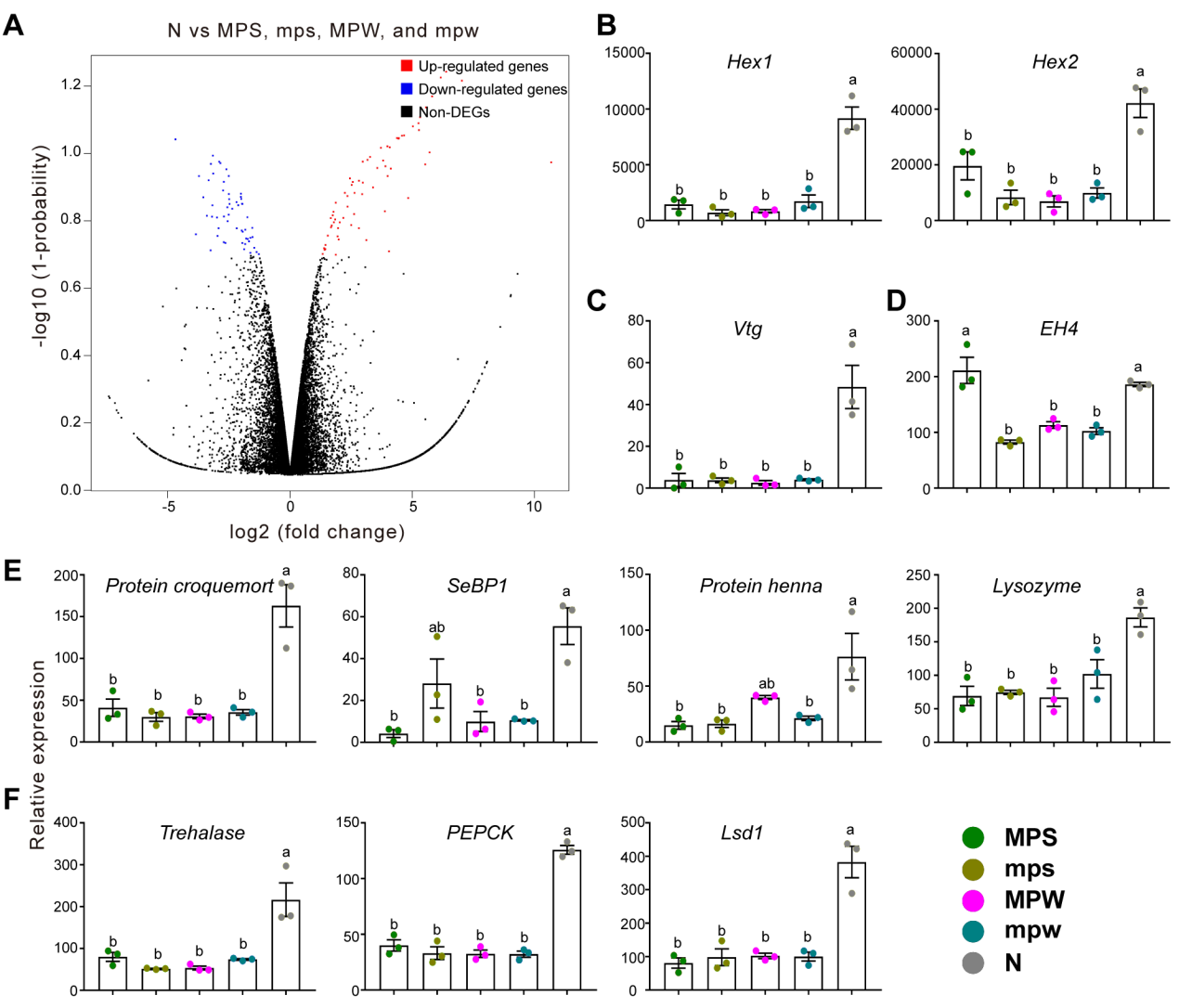
**

**Figure S6. DEGs between nymphs and the other immature castes. (A)** Distribution of gene expression between nymphs and the other immature castes; the expression levels of **(B)** two genes associated with caste differentiation, **(C)** a gene associated with reproduction, **(D)** a gene associated with detoxification, **(E)** four genes associated with immunity, and **(F)** three genes associated with energy metabolism. Error bars represent the mean ± S.E.M. Different lowercase letters over the bars denote significant differences (*P* < 0.05). N, nymphs; MPS, major presoldiers; mps, minor presoldiers; MPW, major preworkers; mpw, minor preworkers.
