# Supplementary material for: Transcriptomic and Functional Analyses of Phenotypic Plasticity in a Higher Termite, Macrotermes barneyi Light
Source: Front Genet. 2019 Oct 4;10:964. doi: 10.3389/fgene.2019.00964 (PMC6797822; doi:10.3389/fgene.2019.00964)
Supplement: Supplementary file 6 [file DataSheet_1.zip › Data Sheet 1/Supplementary Figures and Tables/Figure S8.docx]

**
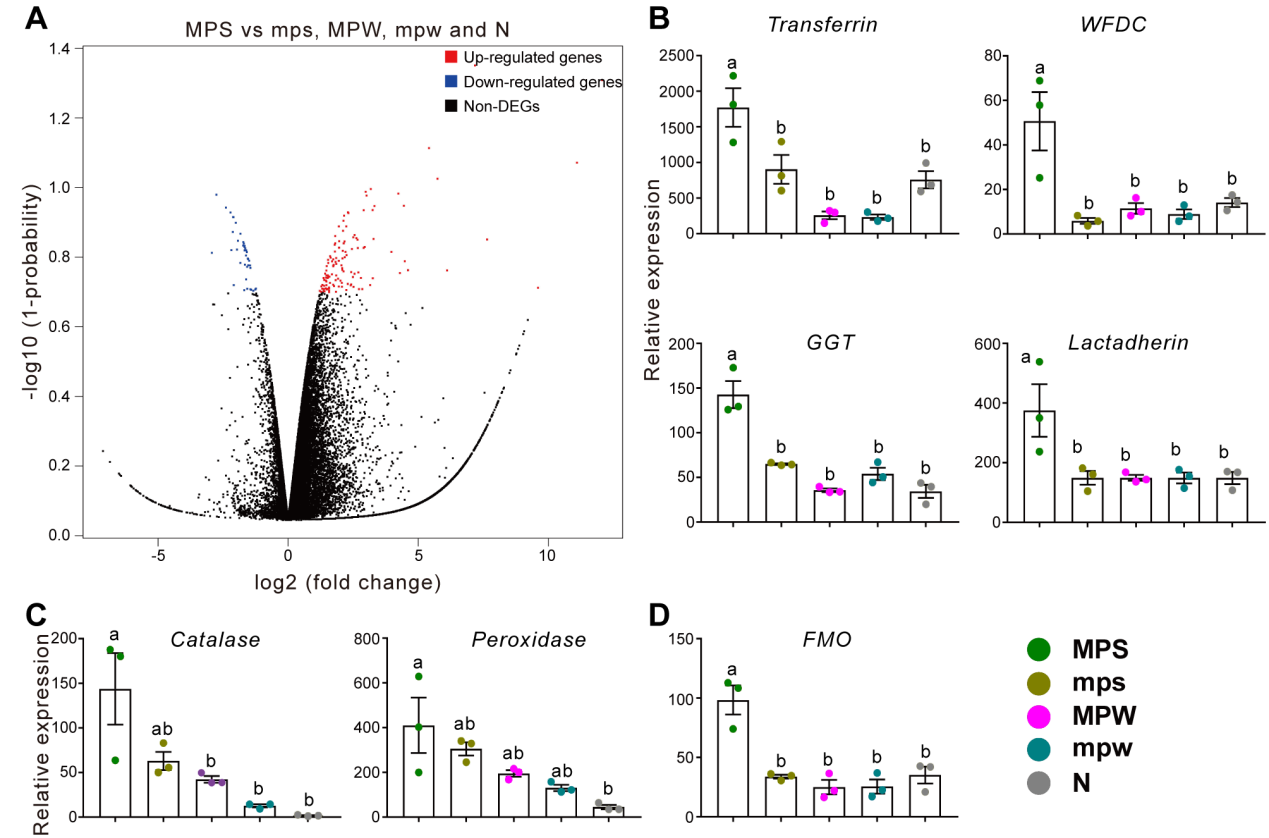
**

**Figure S8.** **DEGs between major presoldiers and the other immature castes. (A)** Distribution of gene expression between major presoldiers and the other immature castes; expression levels of **(B)** four genes associated with immunity, **(C)** two genes associated with antioxidation, and **(D)** a gene associated with detoxification. Error bars represent the mean ±S.E.M. Different lowercase letters over the bars denote significant differences (*P* < 0.05). N, nymphs; MPS, major presoldiers; mps, minor presoldiers; MPW, major preworkers; mpw, minor preworkers.
