# Supplementary material for: Transcriptomic and Functional Analyses of Phenotypic Plasticity in a Higher Termite, Macrotermes barneyi Light
Source: Front Genet. 2019 Oct 4;10:964. doi: 10.3389/fgene.2019.00964 (PMC6797822; doi:10.3389/fgene.2019.00964)
Supplement: Supplementary file 6 [file DataSheet_1.zip › Data Sheet 1/Supplementary Figures and Tables/Table S1.docx]

**Table S1. Locations and collection details of 11 *M. barneyi* colonies.**

| **Colony** | **Location** | **Position** | **Altitude** | **Details of collection** | **Purpose** |
| --- | --- | --- | --- | --- | --- |
| 1 | Shuangfeng county, Hunan province, China | N 27^o^16'29.64"  E 111^o^0'13.5" | 139 m | Nymph, major and minor presoldiers, major and minor preworkers | Illumina sequencing; RT-qPCR validation of DEGs; validation of alternative splicing. |
| 2 | Shuangfeng county, Hunan province, China | N 27^o^16'28.20"  E 112^o^0'13.32" | 162 m | Major and minor presoldiers, major and minor preworkers |  |
| 3 | Shuangfeng county, Hunan province, China | N 27^o^16'28.56"  E 112^o^0'13.32" | 165 m | Major and minor presoldiers, major and minor preworkers |  |
| 4 | Shuangfeng county, Hunan province, China | N 27^o^15'41.76"  E 111^o^35'44.88" | 173 m | Nymph |  |
| 5 | Shuangfeng county, Hunan province, China | N 27^o^15'41.76"  E 111^o^35'46.32" | 165 m | Nymph |  |
| 6 | Wuhan county, Hubei province, China | N 30^o^12'8.9" | 28 m | Nymph | Validation of RNAi targeting *Vtg* in nymph. |
|  |  | E 114^o^17'2.2" |  |  |  |
| 7 | Changsha county, Hunan province, China | N 28^o^6'56.33" | 70 m | Nymph, minor presoldiers, major preworkers | Phenotypic assays of RNAi targeting *Vtg* in nymph; validation of RNAi targeting *TnC* in major prewokers; phenotypic assays of RNAi targeting *TnC* in minor presoldiers and major preworkers. |
|  |  | E 113^o^4'47.33" |  |  |  |
| 8 | Changsha county, Hunan province, China | N 28^o^6'55.47" | 69 m | Minor presoldiers and major preworkers | Validation of RNAi targeting *TnC* in major prewokers; phenotypic assays of RNAi targeting *TnC* in minor presoldiers and major preworkers. |
|  |  | E 113^o^4'47.34" |  |  |  |
| 9 | Changsha county, Hunan province, China | N 28^o^17'47" | 216 m | Minor presoldiers and major preworkers |  |
|  |  | E 112^o^53'45" |  |  |  |
| 10 | Jian county, Jiangxi province, China | N 27^o^26'9.63" | 60 m | Minor presoldiers | Validation of RNAi targeting *TnC* in minor presoldiers |
|  |  | E 114^o^58'11.77" |  |  |  |
| 11 | Jian county, Jiangxi province, China | N 27^o^26'10.01" | 51 m | Minor presoldiers |  |
|  |  | E 114^o^57'56.85" |  |  |  |
