# Supplementary material for: Transcriptomic and Functional Analyses of Phenotypic Plasticity in a Higher Termite, Macrotermes barneyi Light
Source: Front Genet. 2019 Oct 4;10:964. doi: 10.3389/fgene.2019.00964 (PMC6797822; doi:10.3389/fgene.2019.00964)
Supplement: Supplementary file 6 [file DataSheet_1.zip › Data Sheet 1/Supplementary Figures and Tables/Table S10.docx]

**Table S10. Statistics of AS events among the nine targeted comparative groups.**

| **Comparative groups** | **A3SS** | **A5SS** | **MXE** | **RI** | **SE** | **Total** |
| --- | --- | --- | --- | --- | --- | --- |
| **N vs others** | 923 | 689 | 5405 | 302 | 23071 | 30390 |
| **MPS vs others** | 919 | 683 | 5398 | 302 | 23017 | 30319 |
| **mps vs others** | 923 | 688 | 5401 | 303 | 23055 | 30370 |
| **MPW vs others** | 914 | 686 | 5403 | 302 | 23071 | 30376 |
| **mpw vs others** | 921 | 689 | 5407 | 303 | 23060 | 30380 |
| **MPS and mps vs others** | 928 | 692 | 5407 | 304 | 23086 | 30417 |
| **MPW and mpw vs others** | 922 | 692 | 5409 | 303 | 23089 | 30415 |
| **MPS vs mps** | 817 | 624 | 2277 | 291 | 13134 | 17143 |
| **MPW vs mpw** | 816 | 632 | 2342 | 297 | 13602 | 17689 |

**Note:** A3SS, alternative 3' splicing site; A5SS, alternative 5' splicing site; AS, alternative splicing; MXE, mutually exclusive exons; RI, retained intron; SE, skipped exon; N, nymphs; MPS, major presoldiers; mps, minor presoldiers; MPW, major preworkers; mpw, minor preworkers.
