# Supplementary material for: Transcriptomic and Functional Analyses of Phenotypic Plasticity in a Higher Termite, Macrotermes barneyi Light
Source: Front Genet. 2019 Oct 4;10:964. doi: 10.3389/fgene.2019.00964 (PMC6797822; doi:10.3389/fgene.2019.00964)
Supplement: Supplementary file 6 [file DataSheet_1.zip › Data Sheet 1/Supplementary Figures and Tables/Table S11.docx]

**Table S11. Statistics of the genes with significantly different AS events among the nine targeted comparative groups.**

| **Groups** | **A3SS** | **A5SS** | **MXE** | **RI** | **SE** | **Total** |
| --- | --- | --- | --- | --- | --- | --- |
| **N vs others** | 1 | 4 | 6 | 2 | 27 | **41** |
| **MPS vs others** | 9 | 1 | 40 | 5 | 36 | **91** |
| **mps vs others** | 2 | 4 | 28 | 2 | 27 | **63** |
| **MPW vs others** | 2 | 4 | 33 | 4 | 32 | **75** |
| **mpw vs others** | 3 | 1 | 18 | 2 | 25 | **49** |
| **MPS and mps vs others** | 6 | 2 | 40 | 3 | 73 | **122** |
| **MPW and mpw vs others** | 5 | 1 | 38 | 6 | 56 | **106** |
| **MPS vs mps** | 2 | 5 | 24 | 1 | 12 | **44** |
| **MPW vs mpw** | 1 | 6 | 10 | 0 | 10 | **27** |

**Note:** FDR ≤ 0.05. A3SS, alternative 3' splicing site; A5SS, alternative 5' splicing site; AS, alternative splicing; MXE, mutually exclusive exons; RI, retained intron; SE, skipped exon; N, nymphs; MPS, major presoldiers; mps, minor presoldiers; MPW, major preworkers; mpw, minor preworkers.
