# Supplementary material for: Transcriptomic and Functional Analyses of Phenotypic Plasticity in a Higher Termite, Macrotermes barneyi Light
Source: Front Genet. 2019 Oct 4;10:964. doi: 10.3389/fgene.2019.00964 (PMC6797822; doi:10.3389/fgene.2019.00964)
Supplement: Supplementary file 6 [file DataSheet_1.zip › Data Sheet 1/Supplementary Figures and Tables/Table S12.docx]

**Table S12. Function classification of differentially spliced genes among the five immature castes**.

|  | | **Functional classification** | | | | | | |
| --- | --- | --- | --- | --- | --- | --- | --- | --- |
|  |  | **Muscle development** | **Gene activity** | **Cuticle development** | **Energy metabolism** | **Immunity** | **Neural signal** | **Other function** |
| **N vs others** | Gene number | 4 | 9 | 4 | 3 | 0 | 2 | 11 |
|  | AS number | 8 | 9 | 8 | 3 | 0 | 2 | 11 |
| **MPS and mps vs others** | Gene number | 22 | 4 | 0 | 9 | 0 | 9 | 34 |
|  | AS number | 63 | 5 | 0 | 9 | 0 | 9 | 36 |
| **MPS vs others** | Gene number | 10 | 5 | 0 | 2 | 1 | 4 | 27 |
|  | AS number | 33 | 5 | 0 | 13 | 2 | 5 | 32 |
| **MPW and mpw vs others** | Gene number | 10 | 9 | 1 | 4 | 0 | 6 | 29 |
|  | AS number | 48 | 9 | 1 | 8 | 0 | 6 | 33 |

**Note:** AS, alternative splicing; N, nymphs; MPS, major presoldiers; mps, minor presoldiers; MPW, major preworkers; mpw, minor preworkers.
