# Supplementary material for: Transcriptomic and Functional Analyses of Phenotypic Plasticity in a Higher Termite, Macrotermes barneyi Light
Source: Front Genet. 2019 Oct 4;10:964. doi: 10.3389/fgene.2019.00964 (PMC6797822; doi:10.3389/fgene.2019.00964)
Supplement: Supplementary file 6 [file DataSheet_1.zip › Data Sheet 1/Supplementary Figures and Tables/Table S13.docx]

**Table S13. The genome location of the four validated AS events**.

| **Gene name** | **Gene ID** | **Scaffold ID** | **AS type** | **Skip exon start-end** | **Upstream exon start-end** | **Downstream exon start-end** |
| --- | --- | --- | --- | --- | --- | --- |
| ***MHC*** | Mnat_04713 | scaffold165 | SE | 616538-616709 | 610131-610281 | 617445-617673 |
| ***PDZ-LIM domain protein*** | Mnat_02441 | scaffold82 | SE | 1023511-1023568 | 1021373-1021526 | 1029870-1029959 |
| ***Titin*** | Mnat_11129 | scaffold508 | SE | 187382-187430 | 184538-184686 | 188050-188089 |
|  |  |  |  | **Long exon start-end** | **Short exon start-end** | **Flanking exon start-end** |
| ***Tensin*** | Mnat_04364 | scaffold148 | A5SS | 3858375-3858711 | 3858375-3858549 | 3863341-3863544 |

**Note:** AS, alternative splicing; SE, skipped exon; A5SS, alternative 5' splicing site.
