# Supplementary material for: Transcriptomic and Functional Analyses of Phenotypic Plasticity in a Higher Termite, Macrotermes barneyi Light
Source: Front Genet. 2019 Oct 4;10:964. doi: 10.3389/fgene.2019.00964 (PMC6797822; doi:10.3389/fgene.2019.00964)
Supplement: Supplementary file 6 [file DataSheet_1.zip › Data Sheet 1/Supplementary Figures and Tables/Table S2.docx]

**Table S2. Stability rankings of candidate reference genes calculated by geNorm.**

| **Ranking** | **Gene name** | **M value** |
| --- | --- | --- |
| 1 | *HSP70* | 0.031 |
| 2 | *GAPDH* | 0.032 |
| 3 | *Actin* | 0.035 |
| 4 | *EF1-α* | 0.036 |
| 5 | *GST* | 0.042 |
