# Supplementary material for: Transcriptomic and Functional Analyses of Phenotypic Plasticity in a Higher Termite, Macrotermes barneyi Light
Source: Front Genet. 2019 Oct 4;10:964. doi: 10.3389/fgene.2019.00964 (PMC6797822; doi:10.3389/fgene.2019.00964)
Supplement: Supplementary file 6 [file DataSheet_1.zip › Data Sheet 1/Supplementary Figures and Tables/Table S3.docx]

**Table S3. Sequences of primers used in this study.**

| **Gene Name** | **Orientation** | | **(5′→3′) Primer Sequence** | **Purpose** |
| --- | --- | --- | --- | --- |
| *HSP70* | Forward | GAGATTGGTCAGATGCTACAGTTC | | Reference gene validation, reference gene for RT-PCR and RT-qPCR |
|  | Reverse | CCTGCGATGTGGTTACTTCAA | |  |
| *GAPDH* | Forward | CGTATTGGCCGTCTTGTGCT | | Reference gene validation, reference gene for RT-PCR and RT-qPCR |
|  | Reverse | TGAAGCGACCATGGGTGGAA | |  |
| *Actin* | Forward | GGCTGCCTGTAAACACCCTG | | Reference gene validation |
|  | Reverse | GGACCCAACGGCCACATTAC | |  |
| *EF1-α* | Forward | TGCATGGGTGTTGGACAAGC | | Reference gene validation |
|  | Reverse | TCTGTGCCCAGGAGCATCAA | |  |
| *GST* | Forward | AATCTCATGGAGGGGGCACA | | Reference gene validation |
|  | Reverse | GATTGCACGGCTTTCCCACA | |  |
| *Hex1* | Forward | GGAGGAGTGCCAGAACATGA | | RT-qPCR for validation of DEGs |
|  | Reverse | TCGCCAACGACTTCGATACT | |  |
| *Hex2* | Forward | GTCCGTATCGAGCAACCGAA | | RT-qPCR for validation of DEGs |
|  | Reverse | GGAGAGTATGGCACTCCACG | |  |
| *Vtg* | Forward | CAAAAGTTTCGGTCCTGCCG | | RT-qPCR for validation of DEGs and RT-qPCR for RNAi efficiency |
|  | Reverse | GTCGCGTCGTGTCTTCGATA | |  |
| *Lsd1* | Forward | GCTGGTGAATTGGGGACTGA | | RT-qPCR for validation of DEGs |
|  | Reverse | CAGAGTGACCGCTGGTATCC | |  |
| *Transferrin* | Forward | AACTACGCATTCCTCTGCCC | | RT-qPCR for validation of DEGs |
|  | Reverse | TTCTCTCCAATGGTGTCGGC | |  |
| *Catalase* | Forward | GGCAGGTGCTTTCGGTTACT | | RT-qPCR for validation of DEGs |
|  | Reverse | GGCACTCGCTTCCCAATCT | |  |
| *Protein croquemort* | Forward | TACGTCACTTTGGAGCCGTC | | RT-qPCR for validation of DEGs |
|  | Reverse | GCGCACCAGAGTACAGACTT | |  |
| *GGT* | Forward | TGCTTCTGTTGAAGCCGTGA | | RT-qPCR for validation of DEGs |
|  | Reverse | CACCAAGTCTGCGTTGACAG | |  |
| *Homeotic protein deformed* | Forward | CACGAAGAACGTCCGAAGGA | | RT-qPCR for validation of DEGs |
|  | Reverse | TCGACCTCGTTGTTGCGTTA | |  |
| *Flightin* | Forward | CCAGTGATGGATATCGCCTG | | RT-qPCR for validation of DEGs |
|  | Reverse | CGCACGTACACGAAAGACAAG | |  |
| *TnC* | Forward | CCTTCGACGCTTTTGATCGC | | RT-qPCR for validation of DEGs |
|  | Reverse | CTGTTGAAGGGCTGACCCAT | |  |
| *Collagen* | Forward | ACAGTAAACACGGGGGACAC | | RT-qPCR for validation of DEGs |
|  | Reverse | TTGAACCTTTCCCGCCCTTT | |  |
| *PEPCK* | Forward | AGTGTTTATTGGGGCTGCCA | | RT-qPCR for validation of DEGs |
|  | Reverse | CGTGGAACACCTTGGGAAGT | |  |
| *Trehalase* | Forward | AGCAATGATCTGCAGAGGTGG | | RT-qPCR for validation of DEGs |
|  | Reverse | ACTTAGGGGGCATACCAACA | |  |
| *SeBP1* | Forward | TCAGCGATCCGAAGAATCCG | | RT-qPCR for validation of DEGs |
|  | Reverse | TGGTCCATCGCTCTGTATGC | |  |
| *Lysozyme* | Forward | CCAAAACCGTTGGCGTGTTC | | RT-qPCR for validation of DEGs |
|  | Reverse | GATAATGCAGCAGCGCGAAG | |  |
| *MHC* | Forward | GCCAAGTTCCGCAAAGCAC | | RT-PCR for AS analysis |
| Skipped exon | Reverse | ATGGCGTTCACAACCCTCTG | |  |
| *PDZ-LIM domain protein* | Forward | CGCACAACACCACTCGTACT | | RT-PCR for AS analysis |
| Skipped exon | Reverse | TGTGACTGGATTGTCTCGGTGA | |  |
| *Titin* Skipped exon | Forward | TTGCATCCCAGAAGACGGTG | | RT-PCR for AS analysis |
|  | Reverse | TCGAGGTCTCCCTCCTTTGT | |  |
| *Tensin* A5SS | Forward | GCAGCAACAAACTTCCACACT | | RT-PCR for AS events |
|  | Reverse | GGGGATGAACGGGAAATGCT | |  |
| ds*Vtg* | Forward | taatacgactcactatagggTGTTACCCCACGTCACAGAA | | Primers with T7 promoter for dsRNA |
|  | Reverse | taatacgactcactatagggTTGACTTCCACCTCCGTTTC | |  |
| ds*TnC* | Forward | taatacgactcactatagggAAGGCCTTCGACGCTTTTGA | | Primers with T7 promoter for dsRNA |
|  | Reverse | taatacgactcactatagggTTAGCCAGTCATCATCTCCATGAAT | |  |
| ds*GFP* | Forward | taatacgactcactatagggCTTGAAGTTGACCTTGATGCC | | Primers with T7 promoter for dsRNA |
|  | Reverse | taatacgactcactatagggTGGTCCCAATTCTCGTGGAAC | |  |
| *TnC* | Forward | CCTTCGACGCTTTTGATCGC | | RT-qPCR for RNAi efficiency |
|  | Reverse | CTGTTGAAGGGCTGACCCAT | |  |
