# Supplementary material for: Transcriptomic and Functional Analyses of Phenotypic Plasticity in a Higher Termite, Macrotermes barneyi Light
Source: Front Genet. 2019 Oct 4;10:964. doi: 10.3389/fgene.2019.00964 (PMC6797822; doi:10.3389/fgene.2019.00964)
Supplement: Supplementary file 6 [file DataSheet_1.zip › Data Sheet 1/Supplementary Figures and Tables/Table S4.docx]

**Table S4. The data distribution of** **morphological parameters and functional analyses of *Vtg* and *TnC* evaluated by shapiro-wilk test.**

| **Data** | **W** | ***df*** | ***P*** |  |
| --- | --- | --- | --- | --- |
| Body lengths of major presoldiers | 0.852 | 6 | 0.164 |  |
| Body lengths of minor presoldiers | 0.882 | 6 | 0.279 |  |
| Body lengths of major preworkers | 0.888 | 6 | 0.307 |  |
| Body lengths of minor preworkers | 0.871 | 6 | 0.172 |  |
| Head widths of major presoldiers | 0.890 | 6 | 0.177 |  |
| Head widths of minor presoldiers | 0.860 | 6 | 0.165 |  |
| Head widths of major preworkers | 0.917 | 6 | 0.485 |  |
| Head widths of minor preworkers | 0.971 | 6 | 0.898 |  |
| Eclosion rates of nymphs treated with ds*Vtg* for one day^*^ | - | - | - |  |
| Eclosion rates of nymphs treated with ds*GFP* for one day^*^ | - | - | - |  |
| Eclosion rates of nymphs treated with ds*Vtg* for two days | 0.664 | 7 | 0.001 |  |
| Eclosion rates of nymphs treated with ds*GFP* for two days^*^ | - | - | - |  |
| Eclosion rates of nymphs treated with ds*Vtg* for three days | 0.833 | 7 | 0.086 |  |
| Eclosion rates of nymphs treated with ds*GFP* for three days^*^ | - | - | - |  |
| Eclosion rates of nymphs treated with ds*Vtg* for four days | 0.840 | 7 | 0.099 |  |
| Eclosion rates of nymphs treated with ds*GFP* for four days | 0.453 | 7 | 0.000 |  |
| ATP levels of minor presoldiers treated with ds*TnC* | 0.826 | 11 | 0.020 | |
| ATP levels of minor presoldiers treated with ds*GFP* | 0.801 | 11 | 0.010 |  |
| ATP levels of major preworkers treated with ds*TnC* | 0.861 | 8 | 0.123 |  |
| ATP levels of major preworkers treated with ds*GFP* | 0.791 | 8 | 0.023 |  |
| Velocities of minor presoldiers treated with ds*TnC* | 0.942 | 22 | 0.217 |  |
| Velocities of minor presoldiers treated with ds*GFP* | 0.955 | 22 | 0.404 |  |
| Velocities of major preworkers treated with ds*TnC* | 0.959 | 24 | 0.424 |  |
| Velocities of major preworkers treated with ds*GFP* | 0.963 | 24 | 0.501 |  |
| Distances of minor presoldiers treated with ds*TnC* | 0.932 | 22 | 0.136 |  |
| Distances of minor presoldiers treated with ds*GFP* | 0.911 | 22 | 0.049 |  |
| Distances of major preworkers treated with ds*TnC* | 0.955 | 24 | 0.350 |  |
| Distances of major preworkers treated with ds*GFP* | 0.962 | 24 | 0.490 |  |

**^*^Note:** Eclosion rates of nymphs in these groups were zero (0).
