# Supplementary material for: Transcriptomic and Functional Analyses of Phenotypic Plasticity in a Higher Termite, Macrotermes barneyi Light
Source: Front Genet. 2019 Oct 4;10:964. doi: 10.3389/fgene.2019.00964 (PMC6797822; doi:10.3389/fgene.2019.00964)
Supplement: Supplementary file 6 [file DataSheet_1.zip › Data Sheet 1/Supplementary Figures and Tables/Table S5.docx]

**Table S5. The equality of variances evaluated by Leven’s test.**

| **Data** | ***P* value** | **Statistical methods** |  |
| --- | --- | --- | --- |
| ATs densities of *MHC* | 0.053 | One-way ANOVA; Tukey’s HSD test |  |
| ATs densities of *PDZ-LIM domain protein* | 0.253 | One-way ANOVA; Tukey’s HSD test |  |
| ATs densities of *Titin* | 0.083 | One-way ANOVA; Tukey’s HSD test |  |
| ATs densities of *Tensin* | 0.341 | One-way ANOVA; Tukey’s HSD test |  |
| Expression of *Hex1* validated by RT-qPCR | 0.020 | Welch’s ANOVA; Games-Howell test |  |
| Expression of *Hex2* validated by RT-qPCR | 0.051 | One-way ANOVA; Tukey’s HSD test |  |
| Expression of *Vtg* validated by RT-qPCR | 0.004 | Welch’s ANOVA; Games-Howell test |  |
| Expression of *Trehalase* validated by RT-qPCR | 0.061 | One-way ANOVA; Tukey’s HSD test |  |
| Expression of *PEPCK* validated by RT-qPCR | 0.070 | One-way ANOVA; Tukey’s HSD test |  |
| Expression of *Lsd1* validated by RT-qPCR | 0.002 | Welch’s ANOVA; Games-Howell test |  |
| Expression of *Lysozyme* validated by RT-qPCR | 0.035 | Welch’s ANOVA; Games-Howell test |  |
| Expression of *Protein croquemort* validated by RT-qPCR | 0.148 | One-way ANOVA; Tukey’s HSD test |  |
| Expression of *SeBP1* validated by RT-qPCR | 0.007 | Welch’s ANOVA; Games-Howell test |  |
| Expression of *Transferrin* validated by RT-qPCR | 0.009 | Welch’s ANOVA; Games-Howell test |  |
| Expression of *GGT* validated by RT-qPCR | 0.056 | One-way ANOVA; Tukey’s HSD test |  |
| Expression of *Catalase* validated by RT-qPCR | 0.004 | Welch’s ANOVA; Games-Howell test |  |
| Expression of *Flightin* validated by RT-qPCR | 0.016 | Welch’s ANOVA; Games-Howell test | |
| Expression of *TnC* validated by RT-qPCR | 0.002 | Welch’s ANOVA; Games-Howell test |  |
| Expression of *Homeotic protein deformed* validated by RT-qPCR | 0.107 | One-way ANOVA; Tukey’s HSD test |  |
| Expression of *Collagen IV* validated by RT-qPCR | 0.053 | One-way ANOVA; Tukey’s HSD test |  |
| FPKM of *Hex1* | 0.015 | Welch’s ANOVA; Games-Howell test |  |
| FPKM of *Hex2* | 0.067 | One-way ANOVA; Tukey’s HSD test |  |
| FPKM of *Vtg* | 0.053 | One-way ANOVA; Tukey’s HSD test |  |
| FPKM of *EH4* | 0.006 | Welch’s ANOVA; Games-Howell test |  |
| FPKM of *Protein croquemort* | 0.057 | One-way ANOVA; Tukey’s HSD test |  |
| FPKM of *SeBP1* | 0.018 | Welch’s ANOVA; Games-Howell test |  |
| FPKM of *Protein henna* | 0.005 | Welch’s ANOVA; Games-Howell test |  |
| FPKM of *Lysozyme* | 0.381 | One-way ANOVA; Tukey’s HSD test |  |
| FPKM of *Trehalase* | 0.001 | Welch’s ANOVA; Games-Howell test |  |
| FPKM of *PEPCK* | 0.625 | Welch’s ANOVA; Games-Howell test |  |
| FPKM of *Lsd1* | 0.018 | Welch’s ANOVA; Games-Howell test |  |
| FPKM of *Transferrin* | 0.129 | One-way ANOVA; Tukey’s HSD test |  |
| FPKM of *WFDC* | 0.057 | One-way ANOVA; Tukey’s HSD test |  |
| FPKM of *GGT* | 0.007 | Welch’s ANOVA; Games-Howell test |  |
| FPKM of *Lactadherin* | 0.052 | One-way ANOVA; Tukey’s HSD test |  |
| FPKM of *Catalase* | 0.001 | Welch’s ANOVA; Games-Howell test |  |
| FPKM of *Peroxidase* | 0.061 | One-way ANOVA; Tukey’s HSD test |  |
| FPKM of *FMO* | 0.050 | One-way ANOVA; Tukey’s HSD test |  |
| FPKM of *Flightin* | 0.127 | One-way ANOVA; Tukey’s HSD test |  |
| FPKM of *TnC* | 0.009 | Welch’s ANOVA; Games-Howell test |  |
| FPKM of *Acylphosphatase-like protein* | 0.014 | Welch’s ANOVA; Games-Howell test |  |
| FPKM of *SMYD4* | 0.001 | Welch’s ANOVA; Games-Howell test |  |
| FPKM of *Homeotic protein deformed* | 0.052 | One-way ANOVA; Tukey’s HSD test |  |
| FPKM of *Collagen IV* | 0.023 | Welch’s ANOVA; Games-Howell test |  |
| FPKM of *Pro-reslin* | 0.502 | One-way ANOVA; Tukey’s HSD test |  |
| FPKM of *Cuticle protein 8* | 0.002 | Welch’s ANOVA; Games-Howell test |  |
| Body lengths of MPS, mps, MPW, and mpw | 0.002 | Welch’s ANOVA; Games-Howell test |  |
| Head widths of MPS, mps, MPW, and mpw | 0.370 | One-way ANOVA; Tukey’s HSD test |  |
| RNAi efficiency of ds*Vtg* in nymph | 0.331 | Student’s t-test |  |
| RNAi efficiency of ds*TnC* in mps | 0.094 | Student’s t-test |  |
| RNAi efficiency of ds*TnC* in MPW | 0.056 | Student’s t-test |  |
| Velocities of mps treated with ds*TnC* and ds*GFP* | 0.725 | Student’s t-test |  |
| Velocities of MPW treated with ds*TnC* and ds*GFP* | 0.056 | Student’s t-test |  |
| Distances of MPW treated with ds*TnC* and ds*GFP* | 0.055 | Student’s t-test |  |

**Note:** AT, alternative transcript; FPKM, fragments per kilobase million; MPS, major presoldiers; mps, minor presoldiers; MPW, major preworkers; mpw, minor preworkers.
