# Supplementary material for: Transcriptomic and Functional Analyses of Phenotypic Plasticity in a Higher Termite, Macrotermes barneyi Light
Source: Front Genet. 2019 Oct 4;10:964. doi: 10.3389/fgene.2019.00964 (PMC6797822; doi:10.3389/fgene.2019.00964)
Supplement: Supplementary file 6 [file DataSheet_1.zip › Data Sheet 1/Supplementary Figures and Tables/Table S6.docx]

**Table S6.** **The clean data production, quality, GC content and rate of 15 transcriptomes of *M. barneyi*.**

| **Sample Name** | **Sequencing Strategy** | **Read1 GC (%)** | **Read2 GC (%)** | **Read1 Q20 (%)** | **Read2 Q20 (%)** | **Clean Data (bp)** | **Clean Data/Raw Data (%)** |
| --- | --- | --- | --- | --- | --- | --- | --- |
| MPS1 | PE91 | 43.45 | 43.57 | 97.88 | 96.56 | 5,087,506,500 | 94.79 |
| MPS2 | PE91 | 43.37 | 43.47 | 97.91 | 96.64 | 5,082,116,580 | 94.69 |
| MPS3 | PE91 | 45.70 | 45.82 | 97.99 | 96.95 | 5,127,219,000 | 95.53 |
| mps1 | PE91 | 43.55 | 43.68 | 97.81 | 96.80 | 4,992,938,640 | 93.03 |
| mps2 | PE91 | 43.33 | 43.46 | 97.93 | 96.71 | 5,079,414,600 | 94.64 |
| mps3 | PE91 | 44.55 | 44.67 | 98.13 | 97.16 | 5,075,508,960 | 94.57 |
| MPW1 | PE91 | 43.09 | 43.21 | 97.85 | 96.90 | 4,964,533,020 | 92.50 |
| MPW2 | PE91 | 44.21 | 44.33 | 98.05 | 97.37 | 5,058,630,720 | 86.71 |
| MPW3 | PE91 | 43.65 | 43.76 | 98.17 | 97.25 | 4,958,981,640 | 96.60 |
| mpw1 | PE91 | 43.00 | 43.09 | 98.16 | 97.01 | 4,965,824,700 | 92.52 |
| mpw2 | PE91 | 43.78 | 43.91 | 98.14 | 96.96 | 5,024,668,140 | 93.62 |
| mpw3 | PE91 | 43.39 | 43.53 | 97.65 | 96.19 | 5,110,398,360 | 95.22 |
| N1 | PE91 | 42.88 | 43.00 | 97.66 | 96.97 | 4,989,462,660 | 92.96 |
| N2 | PE91 | 45.76 | 45.85 | 98.40 | 97.60 | 4,937,272,380 | 96.18 |
| N3 | PE91 | 45.41 | 45.51 | 97.76 | 97.03 | 5,078,259,000 | 94.62 |

**Note:** The number of reads generated from sequencing after filtering low-quality reads (more than 10% unknown bases; the percentage of no more than Q10 bases is over 50% in a read). N, nymphs; MPS, major presoldiers; mps, minor presoldiers; MPW, major preworkers; mpw, minor preworkers.
