# Supplementary material for: Transcriptomic and Functional Analyses of Phenotypic Plasticity in a Higher Termite, Macrotermes barneyi Light
Source: Front Genet. 2019 Oct 4;10:964. doi: 10.3389/fgene.2019.00964 (PMC6797822; doi:10.3389/fgene.2019.00964)
Supplement: Supplementary file 6 [file DataSheet_1.zip › Data Sheet 1/Supplementary Figures and Tables/Table S7.docx]

**Table S7. Statistics for the mapping of 15 transcriptomes of *M. barneyi* onto the genome of *M. natalensis*.**

| **Sample** | **Total clean reads** | **Mapping rate to genome** | **Mapping rate to genes** | **Total genes number** | **Known genes number** | **Novel genes number** | **Total transcripts number** | **Known transcripts number** | **Novel transcripts number** | |
| --- | --- | --- | --- | --- | --- | --- | --- | --- | --- | --- |
| MPS1 | 56,464,824 | 63.48% | 40.95% | 28816 | 21992 | 6824 | 45094 | 14772 | 30322 |  |
| MPS2 | 56,357,100 | 62.86% | 38.01% | 29500 | 22548 | 6952 | 45700 | 15232 | 30468 |  |
| MPS3 | 54,681,594 | 66.51% | 44.49% | 28886 | 22094 | 6792 | 45612 | 15408 | 30204 |  |
| mps1 | 55,544,078 | 60.85% | 37.35% | 30920 | 23812 | 7108 | 47954 | 16882 | 31072 |  |
| mps2 | 56,460,992 | 57.32% | 37.63% | 30044 | 23000 | 7044 | 46928 | 15936 | 30992 |  |
| mps3 | 56,731,418 | 63.81% | 38.61% | 29896 | 22832 | 7064 | 47104 | 16122 | 30982 |  |
| MPW1 | 55,368,664 | 59.86% | 36.85% | 30890 | 23790 | 7100 | 48070 | 17080 | 30990 |  |
| MPW2 | 56,413,742 | 64.12% | 43.95% | 31138 | 24054 | 7084 | 48444 | 17568 | 30876 |  |
| MPW3 | 55,591,744 | 63.71% | 45.36% | 29722 | 22710 | 7012 | 46676 | 15738 | 30938 |  |
| mpw1 | 55,264,956 | 61.56% | 38.69% | 30688 | 23626 | 7062 | 47920 | 16776 | 31144 |  |
| mpw2 | 55,909,146 | 63.05% | 38.86% | 30352 | 23282 | 7070 | 47764 | 16506 | 31258 |  |
| mpw3 | 56,167,078 | 63.21% | 39.82% | 29796 | 22812 | 6984 | 46718 | 15730 | 30988 |  |
| N1 | 55,461,806 | 61.58% | 37.05% | 30838 | 23786 | 7052 | 47772 | 16754 | 31018 |  |
| N2 | 55,022,012 | 61.66% | 38.15% | 28770 | 21962 | 6808 | 45060 | 14706 | 30354 |  |
| N3 | 54,601,018 | 61.93% | 36.40% | 30154 | 23170 | 6984 | 47724 | 16776 | 30948 |  |

**Note:** N, nymphs; MPS, major presoldiers; mps, minor presoldiers; MPW, major preworkers; mpw, minor preworkers.
