# Supplementary material for: Transcriptomic and Functional Analyses of Phenotypic Plasticity in a Higher Termite, Macrotermes barneyi Light
Source: Front Genet. 2019 Oct 4;10:964. doi: 10.3389/fgene.2019.00964 (PMC6797822; doi:10.3389/fgene.2019.00964)
Supplement: Supplementary file 6 [file DataSheet_1.zip › Data Sheet 1/Supplementary Figures and Tables/Table S8.docx]

**Table S8.** **Significantly enriched GO terms of four comparative groups with the most-abundant DEGs.**

| **Comparative groups** | **GO terms** | **Category** | ***P*-value** |
| --- | --- | --- | --- |
| **mpw vs MPS** | structural molecule activity | Molecular function | 0.01228 |
|  | structural constituent of cuticle | Molecular function | 0.02765 |
| **MPW vs MPS** | structural molecule activity | Molecular function | 0.00038 |
|  | structural constituent of cuticle | Molecular function | 0.00086 |
|  | antioxidant activity | Molecular function | 0.01382 |
|  | peroxidase activity | Molecular function | 0.04742 |
| **N vs mpw** | extracellular space | Cellar component | 0.00721 |
|  | extracellular matrix | Cellar component | 0.03859 |
|  | structural molecule activity | Molecular function | 1.04e-07 |
|  | structural constituent of cuticle | Molecular function | 0.00017 |
|  | structural constituent of chitin-based larval cuticle | Molecular function | 0.01154 |
|  | structural constituent of adult chitin-based cuticle | Molecular function | 0.01154 |
|  | structural constituent of chitin-based cuticle | Molecular function | 0.02293 |
| **N vs MPS** | actin cytoskeleton | Cellar component | 0.00021 |
|  | contractile fiber | Cellar component | 0.01319 |
|  | extracellular space | Cellar component | 0.01754 |
|  | oxidoreductase activity | Molecular function | 0.03060 |
|  | carboxylic acid metabolic process | Biological process | 0.01110 |
|  | oxoacid metabolic process | Biological process | 0.01464 |
|  | organic acid metabolic process | Biological process | 0.01565 |
|  | aromatic amino acid family metabolic process | Biological process | 0.03183 |

**Note:** GO terms with *P*-value < 0.05 are regarded as significantly clustered. N, nymphs; MPS, major presoldiers; mps, minor presoldiers; MPW, major preworkers; mpw, minor preworkers.
