# Supplementary material for: Transcriptomic and Functional Analyses of Phenotypic Plasticity in a Higher Termite, Macrotermes barneyi Light
Source: Front Genet. 2019 Oct 4;10:964. doi: 10.3389/fgene.2019.00964 (PMC6797822; doi:10.3389/fgene.2019.00964)
Supplement: Supplementary file 6 [file DataSheet_1.zip › Data Sheet 1/Supplementary Figures and Tables/Table S9.docx]

**Table S9.** **Statistics of DEGs from the nine targeted comparative groups in the four modules (1, 2, 3, 4).**

| **Comparative groups** | **Modules** | | | |
| --- | --- | --- | --- | --- |
|  | **1** | **2** | **3** | **4** |
| **MPS and mps vs others** | 23 | 10 | 28 | 6 |
| **MPS vs others** | 49 | 35 | 48 | 19 |
| **mps vs others** | 4 | 8 | 0 | 5 |
| **MPS vs mps** | 39 | 49 | 35 | 19 |
| **MPW and mpw vs others** | 26 | 9 | 6 | 14 |
| **MPW vs others** | 6 | 12 | 1 | 9 |
| **mpw vs others** | 36 | 0 | 16 | 12 |
| **MPW vs mpw** | 51 | 29 | 21 | 7 |
| **N vs others** | 10 | 5 | 30 | 59 |

**Note:** N, nymphs; MPS, major presoldiers; mps, minor presoldiers; MPW, major preworkers; mpw, minor preworkers.
